# Evaluating the quality of tabular synthetic data in health care

**DOI:** 10.1371/journal.pdig.0001522

**Published:** 2026-07-07

**Authors:** Ivana Nanevski, Maryam Mohebi, Sebastian Jäger, Karen Otte, Fabian Prasser, Matthias Schulte-Althoff, Daniel Fürstenau, Felix Biessmann

**Affiliations:** 1 Berliner Hochschule für Technik, Berlin, Germany; 2 Berlin Institute of Health at Charité - Universitätsmedizin Berlin, Berlin, Germany; 3 Freie Universität Berlin, Berlin, Germany; 4 Charité - Universitätsmedizin Berlin, Berlin, Germany; 5 Einstein Center Digital Future, Berlin, Germany; Oxford University: University of Oxford, UNITED KINGDOM OF GREAT BRITAIN AND NORTHERN IRELAND

## Abstract

Machine Learning (ML) research in healthcare remains challenging as large, privacy-preserving open datasets are lacking. Synthetic data could offer a solution, but the value of synthetic data depends on diverse and conflicting criteria such as utility, fidelity, and privacy, which are rarely evaluated comprehensively. To close this gap, we explore the trade-off between these metrics in an empirical evaluation across a broad spectrum of generative models, datasets and metrics. In order to include as many metrics and models as possible and to ensure both applicability and comparability with other studies, we focus on the most widely available data modality and task setting: tabular data associated with a classification task. Extending prior work our results demonstrate that no single generative model excels across all metrics and datasets. Across 9 datasets and 11 generative models, the first principal variance direction of all metrics captures the dominant trade-off between fidelity and utility metrics on one side and the privacy metrics on the other side. Sensitivity analyses indicate that the privacy–fidelity/utility trade-off captured by the first principal variance direction remains consistent across several datasets and may support model selection. These insights highlight the potential of synthetic data for responsible data sharing in health care as well as the need for better tooling in synthetic data generation with a higher degree of automation when optimizing for metrics capturing fidelity, utility and privacy.

## Author summary

Artificial Intelligence (AI) has the potential to improve patient treatment with access to high-quality data. However, sharing such data is difficult under strict data protection privacy laws. Many generative AI models have been proposed to produce data that preserves both patients’ privacy as well as the statistical dependency structure and utility of the data. Yet comprehensive empirical evaluations and evaluation protocols that account for all relevant metrics remained scarce. In this study, we present evaluation protocols and experiments comparing generative models for tabular health care data. We assess 11 different generative model architectures for tabular data on 9 datasets to assess three key metrics used in research: fidelity - how good do the synthetic data resemble the original data, utility - how useful these data are, and privacy - how well they protect patient information. Our findings suggest that no single model outperforms all other models in all three metrics. We also find that dataset characteristics, or meta-data, cannot predict the best performing model for a given dataset. But our results also demonstrate that the complexity of the privacy utility trade-off is well captured by the first two principal components. These insights highlight the need for better using another approach - a reliable automation for selecting generative tabular models, especially when the ultimate goal is responsible data sharing in health care.

## 1. Introduction

In recent years, the availability of high-quality public data has become a cornerstone for advancing machine learning (ML) research. This is particularly significant in healthcare research [1,2]. Easy access to medical datasets can significantly advance patient care [3]. However, responsible development of ML models in healthcare must respect patients’ privacy, which requires careful design of data access patterns [4]. Hospitals must navigate strict privacy regulations such as the Health Insurance Portability and Accountability Act (HIPAA) and the European Union General Data Protection Regulation (GDPR) [5], which is essential to protect patient confidentiality [6].

Efforts to mitigate restricted data access and scarcity include approaches like secure processing environments or Federated Learning (FL) [7]. Secure processing environments are often used in practice, here ML researchers usually obtain access to on-premise infrastructure of hospitals after complex legal arrangements. This direct access has substantial disadvantages, such as access to other compute instances, data, code or models is restricted, which slows down model development. Federated Learning implements model training across decentralized data sources without sharing sensitive data, but the technological complexity is usually high for data preparation, prototyping and iterative model development. These are often considered the most time-consuming steps of ML innovations [8,9].

Synthetic data has the potential to offer rapid access to high quality health care data while preserving patients’ privacy. Based on recent advances in generative ML, synthetic data appears to promise responsible ML model development for healthcare, fostering research collaboration while preserving patient confidentiality [3]. For regulatory submissions, they help to replicate analyses and support patient care studies [2,3]. An illustrative example is the study by [10], where synthetic chest Computed Tomography (CT) data improved a COVID-19 related task. Synthetic data can also increase the diversity of datasets and potentially reduce healthcare costs [2,11].

A key challenge with synthetic data is that its responsible usage requires careful empirical evaluations, especially as legal standards for usage of synthetic data in healthcare are not fully specified. To the best of our knowledge, no standardized evaluation framework has been established and widely adopted [6,12].

Even worse, in the current scientific discourse, there is growing ambiguity regarding the metrics for synthetic data evaluation. The authors of [6,13,14] made efforts to classify these based on existing studies, which serve as the foundation to develop a standardized evaluation framework. Building on the insights from all previous research, in this study we focus on the three evaluation metrics: *fidelity*, *utility*, and *privacy*. We explain the metrics considered in this study in detail in Section 2, in short *fidelity* measures how well synthetic data resembles the statistical and structural properties of the original data, *utility* measures how useful the synthetic data is for training ML models on a specific task associated with a dataset, and *privacy* evaluates whether the synthetic data exposes sensitive and confidential patient information.

Several previous studies investigated synthetic data quality metrics [15,16], and frameworks [12,17,18] to evaluate synthetic data. Moreover, despite significant efforts, balancing all three metrics still poses a challenge [12]. For example, in [4,19], the authors have explored balancing utility and privacy using Differential Privacy (DP) [20], a method that adds noise to the data or model outputs to protect individual privacy. However, they highlight the difficulty of balancing privacy and utility, as DP can compromise fidelity and utility without providing significant privacy improvements. There appears to be emerging consensus that more empirical work is needed to assess the value of theoretical concepts such as DP for practical applications in health care [15,16]. The authors of [21] argue that theoretical DP guarantees are not reliable in practice and that disclosure vulnerability must be evaluated empirically. Our study builds on these ideas and provides an empirical evaluation of a broad spectrum of metrics, models and healthcare tabular datasets.

While numerous metrics for synthetic data evaluation have been proposed in the literature [12], their abundance complicates interpretation: different metrics can yield conflicting conclusions [15]. The lack of clear guidelines on selecting the “right” metrics further adds to the challenge [6,13], as optimal choices may vary by dataset and purpose [16]. Researchers emphasize the need for balanced evaluation approaches [6,12], and suggest analyzing diverse models and datasets to establish a general set of metrics [15,16].

We extend previous work on tabular synthetic data in healthcare by considering more datasets and metrics than prior studies, to the best of our knowledge. We present a comprehensive comparison of 11 generative models, covering the most widely used generative model architectures, across 9 benchmark datasets. The quality of the synthetic data is evaluated along three core dimensions - fidelity, utility, and privacy using a suite of 33 evaluation metrics established in the literature.

Our empirical analysis suggests that no single model performs best on all three metrics - fidelity, utility, and privacy. Moreover, we find that dataset meta-features alone cannot predict which model works best for a specific dataset, although this conclusion is limited by the small set of datasets (N = 9) and thus reduced statistical power. We therefore highlight the need an autoML-inspired solution for a more refined selection of generative models for synthetic tabular data.

## 2. Methods

**Fig 1:**
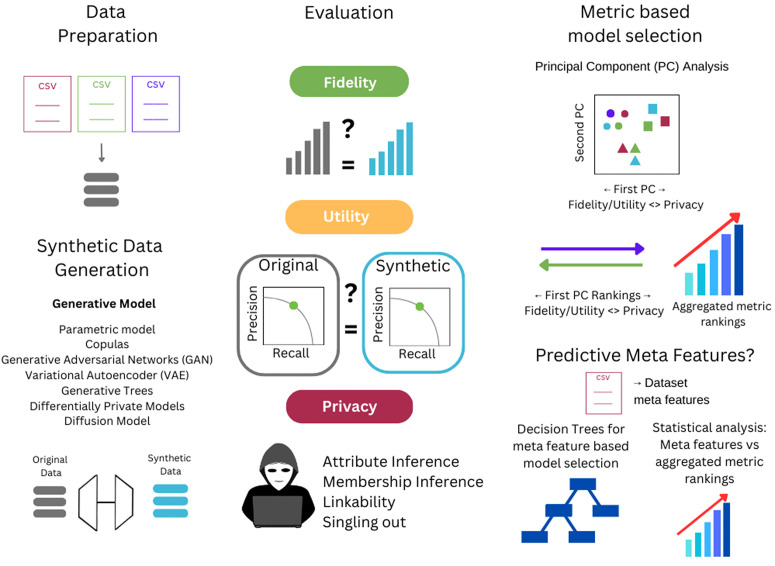
Our framework consists of four steps. After data preparation, we train various generative models to synthesize data. For each generative model, we evaluate fidelity, utility, and privacy of the generated data, and perform several metric-based analyses to guide model selection. Most graphical elements were created by the authors using basic vector shapes. Two icons were obtained from Wikimedia Commons (trapezoid icon in the public domain; attacker illustration released under CC0).

We illustrate the experimental design in Fig 1. First, we preprocess the datasets including mapping categorical variables to interpretable labels or encoding them. Missing data were addressed within the models, where applicable. Next, we generate synthetic datasets using various generative models.

These include Gaussian and Copula based models, Generative Adversarial Networks (GANs), Adversarial Random Forest (ARF), diffusion and differentially private models. Finally, we evaluate the synthetic datasets across three key metrics: fidelity, utility, and privacy. Additionally, we conduct an analysis to explore the trade-off between utility and privacy. For our analyses, we use Synthetic Data Vault (SDV) [22], SDMetrics [23] and Anonymeter [24] to generate the datasets and evaluate their quality. We further evaluate synthetic data by using scores relative to scores computed on original data to provide a clearer contextual comparison. This serves as a worst-case memorization baseline, since we treat the original training data as synthetic data. Specifically, we use the original training split as the “synthetic” dataset and the hold-out test split as the “original” reference dataset, and compute all metrics accordingly. We then compute all metrics for the “synthetic” split relative to the “original” split, which serves as our baseline model. We then compare the actual synthetic data scores to this baseline model scores as a ratio (*relative_score*), which ideally should approach 1, meaning the metrics computed on synthetic data are close to those computed on original data. Finally, we perform multiple metric-based analyses for model selection. We compare our study with similar work as shown in Table 1.

**Table 1:** Comparison with prior empirical benchmarks on synthetic tabular data, summarizing dataset size, evaluated metric families, and model classes.

Study	Datasets	Metric Families	Model Classes
Adams et al. [4]	3	Fidelity, Utility, Privacy	BayesianNet, PrivBayes, PATE-GAN, VAMBN, VAMBN-DP
Dankar et al. [15]	19	Fidelity, Utility	BayesianNet, Gaussian Copula, Synthpop (parametric and nonparametric)
Stadler et al. [19]	2	Utility, Privacy	IndHist, PrivBay, BayNet, CTGAN, PATE-GAN
Yale et al. [25]	1	Fidelity, Utility, Privacy	Gaussian Multivariate, HealthGAN, Parzen Windows, Additive Noise Model, DP data obfuscation, Copy the real data
**Ours**	9	Fidelity, Utility, Privacy	GAN, ARF, VAE, Diffusion, DP generators (PATE-GAN, PrivBayes, DP-WGAN)

### 2.1. Ethics statement

The ethics committee of the Charité – Universitätsmedizin Berlin approved the analysis of Charité and EGZB data (EA2/184/21, Amendment vote Dec 2, 2024). From this analysis, the *University* and *Geriatrics* datasets were derived. Restricted conditions apply. Due to the retrospective nature of the study using standard care data, informed consent was waived. *University* data were anonymized through the institution’s Health Data Platform and subsequent further anonymization steps (e.g., removing irrelevant fields), while EGZB data were anonymized following their internal procedures. The data protection officers and the clinical trial office of the Charité (CTO-22–0838), for the *University* dataset, and, for the *Geriatrics* dataset, the EGZB advised on data protection rules to ensure compliance with those rules. Federal data protection officers were consulted for their advice.

### 2.2. Data

This study is based on 9 datasets (Table 2) from 6 public and 3 proprietary domains to evaluate synthetic data generation methods.

**Table 2:** Detailed summary of all 9 datasets.

	Dataset Name								
Attribute	Diabetes	AIDS	MIMIC-IV-Ext -Fall-Prediction	Geriatric	University	Adult	Heart Failure	Machiavellianism Test	Hospital Discharge
**# Features**	22	23	67	103	45	15	36	16	18
**Sample Size**	229,474	50,000	49,029	12,773	50,000	48,842	1,187	73,489	50,000
**Missing Data**	No	No	Yes	Yes	Yes	No	Yes	No	No
**# Numeric Col.**	3	12	19	24	2	6	9	1	6
**# Integer Col.**	3	11	9	24	2	5	–	–	–
**# Continuous Col.**	0	1	10	0	0	1	–	–	–
**# Boolean Col.**	14	7	27	66	34	1	11	0	0
**# Categorical Col.**	5	4	21	13	9	8	4	15	12
**Target**	Diabetes	Infected	Fall	Fall	Fallen	Income	Heart Failure Risk	Race	Race
**Pos. Label Rate**	15.3%	31.1%	13.47%	13.53%	1.12%	24%	66.4%	1.51%	21.73%
**Missing Rate**	–	–	1.6%	4.7%	38.4%	–	–	–	–
**Dataset type**	Medical	Medical	Medical	Medical	Medical	Socio-economical	Medical	Medical	Medical
**Task type**	Medical	Medical	Medical	Medical	Medical	Socio-economical	Medical	Socio-demographic	Socio-demographic

**Diabetes:** The Diabetes dataset (https://www.kaggle.com/datasets/akshaydattatraykhare/diabetes-dataset) is derived from the 2015 Behavioral Risk Factor Surveillance System (BRFSS) survey. It includes health indicators relevant to diabetes risk, the target feature, with a range of demographic and lifestyle attributes. Licensed under the CC0 Public Domain, this dataset allows unrestricted access for research.

**AIDS Virus Infection:** The AIDS dataset (https://www.kaggle.com/datasets/aadarshvelu/aids-virus-infection-prediction) includes data on HIV/AIDS infections for binary classification tasks. It comprises 23 health-related attributes with Infected being its target feature. Like the Diabetes dataset, is distributed under the CC0 Public Domain license.

**MIMIC-IV-Ext-Fall-Prediction:** For our research purposes, we derive a dataset from MIMIC-IV [26] and include 67 demographic and clinical features. We derive the target column representing inpatient fall incidents based on the patients’ diagnoses. Particularly, we filtered for diagnoses which contained “fall,” but excluded incompatible variants of fall for this task (see Listing A in S1 Appendix for more details). We refer to this dataset as MIMIC-IV-Ext-Fall-Prediction, following their naming conventions for derivatives of the dataset. Access to MIMIC-IV is restricted under the PhysioNet Data Use Agreement (DUA), requiring researchers to complete training and adhere to HIPAA guidelines.

**Geriatric Hospital:** The proprietary Geriatric Hospital dataset was curated for developing a fall risk assessment model. It includes 103 features of mixed types, with missing values reflecting real-world data limitations. Due to its proprietary status, access is restricted. The ethics approval for using this dataset for the purpose of this study is declared in Section 2.1.

**University Hospital:** The proprietary University hospital datasets were also curated for training a fall risk assessment model. It features 45 features tailored for binary fall risk classification. This dataset is also restricted for internal research to ensure patient confidentiality. The ethics approval for using this dataset for the purpose of this study is declared in Section 2.1.

**Adult:** The Adult dataset (https://archive.ics.uci.edu/dataset/2/adult), from the 1994 U.S. Census, contains demographic and income-related data widely used in benchmarking, with income being its target feature. It comprises 15 attributes and is licensed under CC BY 4.0, allowing open access for research.

**Hospital Discharge:** This dataset is a subset of the Texas hospital discharge dataset from 2013 containing 18 attributes describing patient demographics, disease severity state as well as costs associated with their hospital stay. The dataset is accessible as a public use file and can be requested from the Texas Department of State Health Services (https://www.dshs.texas.gov/center-health-statistics/texas-health-care-information-collection/health-data-researcher-information/texas-hospital-emergency-department-research-data-file-ed-rdf/hospital-discharge-data-public-use-data-file).

**Heart Failure:** The Heart Failure dataset includes routine data [27] from partner hospitals of the HiGHmed [28] consortium and contain 36 variables used to calculate the Bio Heart Failure (BIOHF) [29] and the Meta‐Analysis Global Group in Chronic Heart Failure (MAGGIC) [30] risk scores. We used the synthetized version of the dataset [31], which can be accessed upon reasonable request for research purposes.

**Machiavellianism Test:** The Machiavellianism Test dataset is a psychological test dataset to test about machiavellianism. The dataset contains 16 columns related to peoples demographic information and their answers to questions of the test. The data can be openly accessed from the psychometrics project (https://openpsychometrics.org/_rawdata/).

### 2.3. Models

In our evaluation we use 11 different models that represent a spectrum of commonly used generative methods for tabular data. For most models, we use the implementations provided by the SDV package [22]. Only for TabDiff, we follow the implementation of [32], for PATE-GAN and DP-WGAN, the implementation of [33], and for PrivBayes, the implementation of [34]. For most models, we used the recommended hyperparameters provided by the respective implementations without additional tuning. For the DP-based models, we treated the privacy parameter ϵ as a tunable hyperparameter and selected it according to the procedure described in the corresponding sections. Additionally, for DP-WGAN we tuned the number of epochs and learning rates.

#### 2.3.1. Conditional Tabular Generative Adversarial Network (CTGAN).

The CTGAN [35] is designed for generating synthetic tabular data containing mixed data types. It is built upon existing GAN techniques and incorporates a conditional vector and a training-by-sampling approach, which enables it to learn dependencies between categorical and continuous variables and to handle imbalanced categorical distributions.

#### 2.3.2. Gaussian Copula.

The Gaussian Copula model [22] leverages classical statistical methods to synthesize data by modeling feature dependencies with Gaussian copulas. The synthesizer converts all non-numerical features into numerical ones, then transforms the values of each numerical column by mapping them to their cumulative distribution function (CDF) values. These are subsequently converted to a standard normal distribution using an inverse CDF transformation. The model then samples from a multivariate standard normal distribution with the learned correlations.

#### 2.3.3. CopulaGAN.

In [22], CopulaGAN builds upon CTGAN by incorporating a Gaussian Copula. The synthesizer first transforms the features as done in Section 2.3.2, and afterwards a CTGAN is applied to model the correlations between the columns.

#### 2.3.4. Tabular Variational Autoencoder (TVAE).

TVAE [35] adapts the Variational Autoencoder (VAE) architecture for mixed-type tabular data generation. Like with CTGAN, in this study we use TVAE with conditional sampling to maintain the class distributions.

#### 2.3.5. Gaussian Multivariate.

The Gaussian Multivariate synthesizer [36] is implemented using the Copulas library [37] and serves as a simpler copula-based baseline model. Similar to the Gaussian Copula synthesizer Section 2.3.2, it captures feature dependencies through a Gaussian copula, but the main difference is in the data processing. In this approach, a label encoding with added noise is used for transforming non-continuous data. Then the feature dependencies are captured by a Gaussian Copula, enabling the generation of synthetic data by sampling from the multivariate normal distribution.

#### 2.3.6. Wasserstein Generative Adversarial Network (WGAN).

WGAN [38] addresses common GAN limitations, such as mode collapse and training instability, by implementing the Wasserstein distance as a loss function. This approach ensures stable training and reliable gradient signals for the generator. WGAN also adds a gradient penalty to enforce the 1-Lipschitz constraint, further stabilizing the training process.

#### 2.3.7. Adversarial Random Forest (ARF).

ARF [39] combines the generative capabilities of Random Forests with adversarial training to synthesize tabular data. The model iteratively improves synthetic data quality by training a Random Forest to distinguish between real and synthetic data. Synthetic data is generated by sampling observations from the trained forest’s leaves. ARF is computationally efficient and requires minimal tuning, making it a robust alternative for datasets with mixed feature types.

#### 2.3.8. TabDiff.

TabDiff [32] is a diffusion framework designed for mixed-type tabular data. The authors address handling high heterogeneity across different feature distributions with the introduction of a mixed-type feature-wise learnable diffusion processes which simultaneously perturb and denoise numerical and categorical features within a single model.

#### 2.3.9. Private Aggregation of Teacher Ensembles-GAN (PATE-GAN).

PATE-GAN [40] is a differentially private generative adversarial network that combines the Private Aggregation of Teacher Ensembles (PATE) framework with GANs to synthesize privacy-preserving synthetic data. The original dataset is first divided into *k* disjoint partitions, and a separate teacher discriminator is trained on each partition. During training, the generator produces synthetic samples that are evaluated by the teacher discriminators as either “real” or “fake”. These predictions are aggregated with added noise to preserve privacy and are then used to train a student discriminator. Finally, the generator is trained against the student discriminator to generate the synthetic data. Throughout training, the overall privacy budget is tracked under the (ϵ,δ)-differential privacy framework, where ϵ, the privacy budget, controls the privacy loss associated with an individual record, and δ allows a small probability of deviation from the pure ϵ-DP guarantee. For most hyperparameters, we followed the parameterization suggested in [41]. We used a target privacy budget of ϵ∈[0.5,2.0] with $\delta =10^{-5}$. We set both the teacher and student iterations to 5. The private training set of size *N* was partitioned across T=round(N/1000) teacher discriminators, resulting in approximately *N*/*T* samples per teacher partition. Teacher aggregation used the Laplace mechanism with noise parameter $\lambda =10^{-3}$ and training was stopped once the spent privacy budget reached the target ϵ. The final per-dataset ϵ values were selected based on downstream utility performance on the corresponding predictive tasks. We used ϵ=0.7 for the Geriatric dataset, ϵ=1.3 for AIDS and the University datasets, ϵ=1.5 for Diabetes and Adult, and ϵ=2.0 for MIMIC-IV-Ext-Fall-Prediction. The details on the other of hyperparameters are provided in Table A in S1 Appendix.

#### 2.3.10. Differentially Private Wasserstein Generative Adversarial Network (DP-WGAN).

DP-WGAN [42] is a differentially private WGAN trained using gradient norm clipping and additive Gaussian noise applied to the discriminator gradients. We used an (ϵ,δ) DP, with a target privacy budget of ϵ∈[0.5,2.0] with $\delta =10^{-5}$. Based on the per-dataset downstream task performance, we selected ϵ=1.3 for the Geriatric dataset, ϵ=0.7 for MIMIC-IV-Ext-Fall-Prediction, ϵ=1.0 for AIDS, ϵ=1.5 for the University dataset and ϵ=2.0 for Adult and Diabetes. The details on the other of hyperparameters are provided in Table A in S1 Appendix.

#### 2.3.11. PrivBayes.

PrivBayes [34] is a differentially private Bayesian network method. PrivBayes uses differentially private noisy marginal counts to learn a Bayesian network of conditional dependencies between features, and then samples synthetic data from the resulting model. We used the implementation from DataSynthesizer [43].

Due to computational challenges, for datasets with multiple high cardinality features, such as MIMIC-IV-Ext-Fall-Prediction, we use the 30 most common values per feature, and set the rest to OTHER.

### 2.4. Metrics

Quality metrics for synthetic data that capture all legally relevant aspects are an active topic of research [15,16,21]. Previous work has primarily distinguished between utility and privacy [15,16], where utility included metrics which measure fidelity. Fidelity measures how similar synthetic and real datasets are [6,15]. Utility assesses how useful synthetic data is for training a ML model on a downstream task. Privacy is the most critical metric in healthcare data [44] and measures the risks of exposing patients’ identity or their personal information. In this study, we aim at evaluating a broad spectrum of metrics for fidelity, utility, and privacy. To ensure replicability, we use well maintained libraries, such as SDMetrics [23], and extend these where needed with metrics computed using open source libraries, such as Autogluon [45] (for fidelity and utility), scikit-learn [46] and XGBoost [47] (for fidelity), and Anonymeter [24] (for privacy). We match the metrics with other studies, where possible (Tables B, C and D in S1 Appendix). All metrics are shown in Fig A in S1 Appendix.

#### 2.4.1. Fidelity.

We evaluate fidelity using the standard metrics provided in the SDMetrics library [23] and extend these with the Propensity Score as summarized in Fig A in S1 Appendix. The SDMetrics are matched with existing metrics common in the literature (which may have different names) and studies that have used them, as detailed in Table B in S1 Appendix. Fidelity measures the similarity between synthetic and original data [6,15]. More concretely, fidelity metrics measure similarity of single attributes/columns (univariate fidelity), the similarity of second order statistical moments (bivariate fidelity), such as correlations of pairs of attributes, and similarity of the entire distribution of all attributes (multivariate fidelity) [6,15]. To that end, various statistical measures are computed and compared, including non-parametric as well as parametric models.

For univariate fidelity, common approaches include comparing variable distribution [48,49], statistical tests [18,50], and distance-based metrics [15]. In our case, in SDMetrics [23] these metrics include Column Shapes (Kolmogorov-Smirnov Complement and Total Variation Complement), Kullback–Leibler Divergence and Chi-squared test. Bivariate include pairwise feature correlations [15,51], and visualizations such as correlation matrices [17,25]. With SDMetrics [23] this is measured with Column Pair Trends.

To better capture the multivariate similarity of synthetic and real data accounting for the entire data distribution beyond second order statistics, we leverage the *Propensity Score*, or *Distinguishability Performance*, evaluating how well a ML model can distinguish the synthetic data from the original data on held out test data [4,15,16,18,52]. We use the Data Likelihood metrics and the Propensity score from SDMetrics [23] to measure this. In addition, New Row Synthesis [23], or the Novelty Test checks whether the generative model generates new, unique records [18,53].

#### 2.4.2. Utility.

Utility assesses how useful synthetic data is for training ML models. The most common approach is to train two models: one on the original data and another on the synthetic dataset and evaluating them on a test set from the original data (Training on Real, Testing on Real (TRTR) and Training on Synthetic, Testing on Real (TSTR)). Close performance scores of both models mean the synthetic data can be a good proxy for the original data [4,15,16,25,54]. In this study, we use AutoGluon [45], a well maintained AutoML library featuring most ML model classes and end-to-end hyperparameter optimization, to evaluate utility. The original dataset is split into training and test sets, with the test set reserved for validation. We train one model on the original data and another on the synthetic data, both validated using held-out data from the original dataset. We refer to this approach Model Training and Evaluation (commonly known as TRTR and TSTR), and comparing their performances. Comparing predictive performance include Accuracy, F1-score, Precision, and Recall. High-utility synthetic data should show similar predictive scores to the original data. Corresponding metrics (though sometimes named differently), and related studies that have used them are listed in Table C in S1 Appendix.

#### 2.4.3. Privacy.

Privacy is the most critical metric in healthcare data [44] and is evaluated using various approaches, many emulating different attack scenarios. Such attacks may include 1) re-identification attacks, where individual records or groups are matched between synthetic and real-world data to re-identify an individual, 2) attribute inference attacks, where adversaries try to gain information about unknown attributes of a target from the synthetic data, 3) membership inference attacks [55], where adversaries try to determine whether an individual was in the training data, and 4) data linkage attacks, where adversaries use the synthetic dataset to identify whether two disjoint datasets containing attributes from the original data belong to the same individual. In addition, researchers often use distance-based metrics compare synthetic and original records, with metrics such as Distance to Closest Record (DCR) [17,54], and Nearest Neighbor Distance Ratio (NNDR) [17] to quantify information leakage.

In this study, we use five types of privacy metrics: distance based metrics, privacy against inference (or attribute inference attacks), singling out, linkability and membership inference attacks. Each metric covers a different aspect of privacy protection, ensuring a comprehensive evaluation and connect our selection to prior work in Table D in S1 Appendix.

Distance metrics: The distance metrics evaluate privacy risks by measuring the distance between the original and synthetic data. Two metrics are used in this analysis: *Distance to Closest Record (DCR)* [17,54], which measures the Euclidean distance between each synthetic data point and the closest original data point, and *Nearest Neighbor Distance Ratio (NNDR)* [17], which compares the distance between each synthetic data point and its nearest original data point with the distance to its second-nearest original data point. The key outputs for DCR include the 5th Percentile of all DCRs, which is computed for the following dataset combinations: R&S (real and synthetic data), RR (within real data), and SS (within synthetic data). R&S represents the proximity of the closest 5% of synthetic data points to real data points, and the Mean DCR, which provides the average DCR values between both synthetic and original data. A lower 5th percentile DCR R&S suggests potential privacy risks, as it indicates that a subset of the synthetic data points may closely resemble real data. Similarly, NNDR is computed for the following dataset combinations: R&S (real and synthetic data), RR (within real data), and SS (within synthetic data). The primary output for NNDR is the 5th Percentile NNDR R&S, which identifies the closest 5% of cases. A high 5th percentile NNDR indicates that even the nearest synthetic data points maintain a sufficient distance from real data, reflecting stronger privacy protection. For both DCR and NNDR values, the larger the distance, the lower the privacy risk.

**Fig 2:**
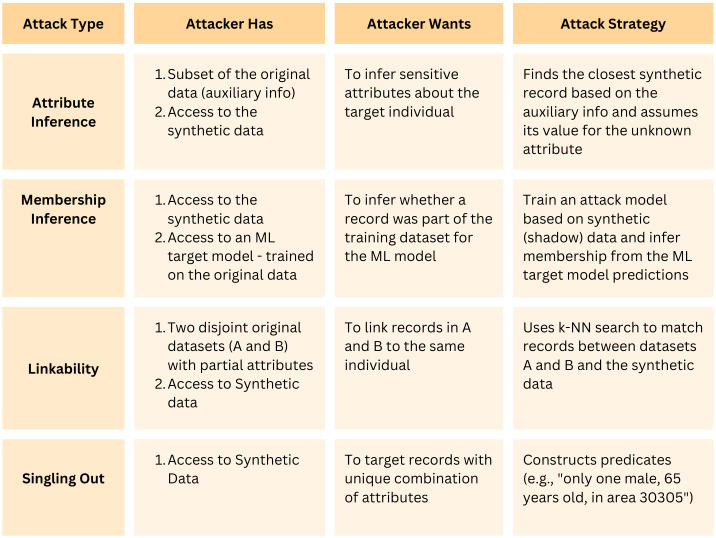
Overview of privacy attack models, listing the attacker’s knowledge (Attacker has), objectives (Attacker Wants), and their Attack Strategy to gain information.

Privacy risk attacks: Evaluating privacy risk attacks on synthetic data provides insights into the level of protection it offers against privacy risks such as attribute inference, membership inference, linkability, and singling out (Fig 2). These insights are important, as the GDPR mandates that any effective anonymization technique (synthetic data in our case) must mitigate these risks to ensure strong data privacy protections [24].

**Membership Inference Attacks:** Membership inference attack (MIA) [55] is a privacy attack in which an adversary attempts to determine whether a specific record was part of the training dataset of a machine learning model. In the context of synthetic data, MIAs aim to infer whether a record was used to train the synthetic data generator, and are difficult to evaluate for generative methods [4,56]. Therefore, we adopt a downstream-task threat model as a practical proxy for membership leakage, assessing whether the released synthetic data holds sufficient auxiliary information to enable membership inference on the original training records via a shadow-based black-box attack.

The assumption of such threats may be realistic in scenarios where synthetic data are released together with, or later used to train, accessible prediction services, benchmark models, or deployed clinical decision-support systems. An alternative attack scenario could involve an adversary that trains a black-box model on the synthetic data, mimicking the downstream black-box model.

To assess this privacy risk, we perform a classic shadow-based black-box membership inference attack and quantify its success using the Privacy Gain (PG) [19], defined through the true positive rate (TPR) and false positive rate (FPR):


$$\begin{array}{c} PG=1-(TPR-FPR) \end{array}$$

$$\begin{array}{c} \text{where}\;TPR=\mathrm{Pr}(s_{t}=1|s=1),\;FPR=\mathrm{Pr}(s_{t}=1|s=0) \end{array}$$

Here, *s* = 1 denotes a member record originating from the original training split (used to train the synthetic data generator), while *s* = 0 denotes a non-member record from the original test split. The quantity TPR-FPR corresponds to the attacker’s advantage, such that higher PG values indicate stronger privacy protection. We assume a realistic threat model in which the attacker has access to the released synthetic data and black-box access to a target (downstream) model trained on the real data. The synthetic samples are used as auxiliary data to train multiple shadow models that approximate the input–output behavior of the target model. Predictions from these shadow models on their respective training and holdout splits are then used to train an attack classifier to distinguish member from non-member records. For each dataset, we train *max*(10, *N*/2000) shadow models, where N denotes the dataset size. Increasing the number of shadow models generally improves attack stability and performance. However, we limit this number to balance the performance with computational cost [55]. Finally, the trained attack model is evaluated on the real private dataset to quantify the resulting privacy gain. For MIA we use the Adversarial Robustness Toolbox [57], also supported in Synthius [36].

**Linkability and Singling Out**: Linkability and singling out [5] are privacy-related metrics whose evaluation is implemented in the Anonymeter framework [24]. Linkability is assessed under the assumption that there are at least two external datasets available to the attacker, that contain some of the original data attributes, and that these attributes are also present in the synthetic data. A low linkability score indicates high privacy. From a patient’s perspective, a “successful” linkability attack confirms that if patient’s age, gender, and area code from one dataset match a record in another dataset containing sensitive information, could potentially expose their private data.

Singling out evaluates the risk of identifying specific individuals in the original dataset based on unique combinations of attributes present in the synthetic data. This risk arises when rare or unique attribute patterns in synthetic data closely match records in the original data. Singling out is assessed through privacy attacks using targeted queries: univariate queries focus on a single attribute (e.g., age), and multivariate queries combine multiple attributes (e.g., age, gender, and location). The intuition behind this approach is that attributes, or their combinations, that are rare or unique in the synthetic data are likely to be rare or unique in the original data as well. In the evaluation phase of the singling out attacks, an attacker’s guess is considered correct if there is exactly one corresponding individual in the original dataset. Again, the closer the success rate of the main to the control or baseline attack, the better. A low singling out score indicates high privacy. From a patient’s perspective, a “successful” singling out attack confirms that there is only one female, X years old patient that lives in area 10188.

**Attribute inference attacks**: Attribute inference attacks [5] pose a threat to patient privacy by enabling attackers to deduce sensitive information from synthetic datasets. Studies such as [55,58] have shown how AI models can be vulnerable to these types of attacks. In this study, we use *(a) Privacy Against Inference (PAI)* from SDMetrics as well as *(b) Attribute Inference Attack* from the Anonymeter framework, to assess attribute inference. (a) PAI evaluates the risk of sensitive attribute disclosure under the assumption that an attacker has access to a subset of the original data. In our experiments we define a set of sensitive attributes for each dataset (Table 3). To perform the attack, the attacker may use different models, such as k-nearest neighbors (KNN), Naive Bayes (NB) classifier, Random Forests (RF) or Support Vector Machine (SVM), and ensemble of classifiers, as well as variations of the Correct Attribution Probability (CAP) algorithm. The higher the scores on these models, the lower the privacy risks. (b) With the attribute inference attack we perform as many attacks as there are attributes in a dataset - N, and not only on a predefined set of sensitive attributes. The goal is to treat every attribute as a sensitive attribute at one point, and based on the N attacks, we choose the highest risk score as a single point estimate for the attribute inference attacks. From a patient’s perspective, a successful” attack could mean that if a patient is X years old, and lives in area Y, an attacker could infer that they are likely to have a risk-factor Q.

**Table 3:** Sensitive attributes considered across datasets. For Heart Failure, copd indicates the presence of Chronic obstructive pulmonary disease, hf_duration represents the heart failure duration in months, hf_gt_18_months indicates whether the heart failure duration is longer than 18 month, and nyha refers to the New York Heart Association score, indicating heart failure severity. Q4A captures the degree of agreement with the statement: “Most people are basically good and kind”.

Dataset	Sensitive attribute
Diabetes	gender, age, income
AIDS	gender, age
MIMIC-IV-Ext-Fall-Prediction	gender, age, race
Geriatric	gender, age
University	gender, age
Adult	gender, race
Heart Failure	smoking, diabetes, copd, hf_duration, hf_gt_18_months, nyha
Machiavellianism Test	Q4A
Hospital Discharge	race, risk_mortality, illness_severity

## 3. Results

We perform an exhaustive evaluation on fidelity, utility, and privacy using 9 datasets and 11 generative models. Due to computational load and operational challenges, we were unable to synthesize data with all models for Heart Failure, Machiavellianism Test, and Hospital Discharge. In the earlier stages of the experimental phase we included TabPFGen [59]. However this model is not included in the analyses due to the lack of sample diversity in the generated data. More specifically, we mostly saw redundant records of two distinctive rows, no novelty and in many cases we noticed that only one class from the target label was produced.

We start the assessment with 33 metrics, 13 of which represent fidelity, 19 describe privacy, and one - F1 macro is our utility metric. To compare models, we aggregate metrics where appropriate across models or datasets, and metrics. We provide all scores in Tables E, F, G, H, I, J, K, L and M in S1 Appendix. For the sake of interpretability, we transform all metric scores where needed, such that higher values mean better scores.

### 3.1. No generative model excels for all datasets

**Fig 3:**
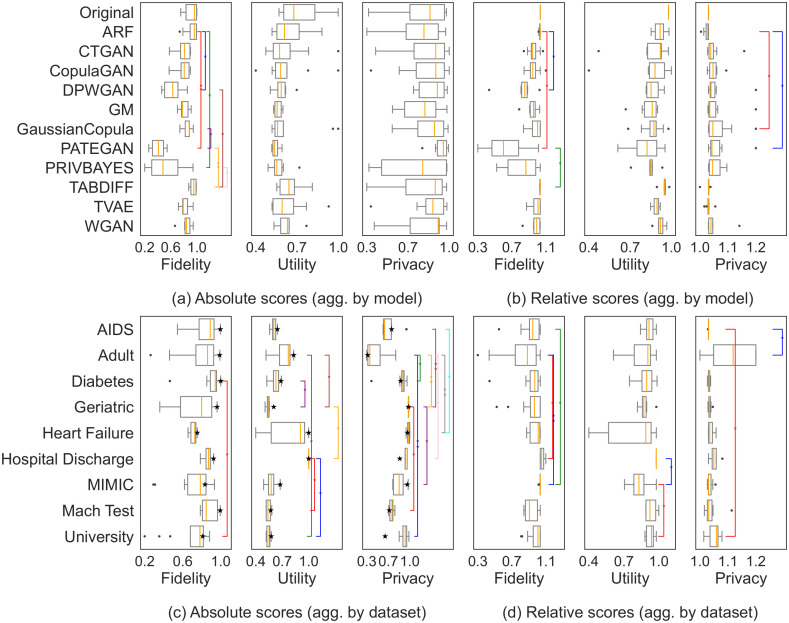
Overview of absolute metrics’ scores (*a,c*) and scores relative to original data (*b,d*) aggregated by fidelity, utility and privacy. We abbreviate Machiavellianism Test as Mach Test, MIMIC-IV-Ext-Fall-Prediction as MIMIC, and GaussianMultivariate as GM for better readability. Comparing metrics across generative models (*a, b*), no model outperforms other models in all metrics. In contrast, comparing metrics across datasets (*c, d*), we see larger and significant (p-value<0.05) differences between datasets. In panel (c), the experiments based on the original data are marked with an asterisk. For (b) and (d), values above 1.25 are capped at 1.25.

In Fig 3 we present the aggregated (median) metrics, fidelity, utility and privacy, across models (panels a and b) and datasets (panels c and d). In panels a and c the absolute values of the aggregated metrics are presented, while in panels b and d we normalize these relative to the metric scores of the original data, as explained in Section 2.

In Fig 3(a), we show that no model dominates across all three metrics. While some models exhibit positive outliers for specific datasets and metrics, such as utility, we do not observe consistent trends across all three metrics.

Comparing all metrics aggregated by dataset, Fig 3(c), the variance is greater between datasets than within datasets, especially in terms of privacy (p-value<0.05). This suggests no clear dominance of any model over a specific dataset. Moreover, to investigate the impact of these differences, we compare these scores to the original data Fig 3(b) and Fig 3(d), where these differences are even less pronounced. To support these findings, we perform pairwise Dunn tests with Bonferroni correction for all three evaluation dimensions: fidelity, utility, and privacy. For the statistical analysis, we use the aggregated median fidelity, utility, and privacy scores computed for each dataset-model pair, resulting in a 9×11 matrix (9 datasets × 11 models) per metric. To compare datasets across each metric (fidelity, utility, and privacy), we use the eleven model scores available per dataset (*N* = 11).

We emphasize that each dataset-model pair was evaluated only once (single training run without repeated random seeds or different hyperparameters). However, we repeated a couple of multiple-seeds experiments for two representative datasets - Adult and AIDS. The results from the different runs align with our main findings. More details will follow in Section 3.2.

We find most statistically significant differences at α=0.05 between datasets in all fidelity, utility and privacy and some models differ in fidelity, as also specified with vertical colored lines in Fig 3. For details, we refer to Tables N, O, P and Q in S1 Appendix.

### 3.2. Exploring metrics to guide model selection

**Fig 4:**
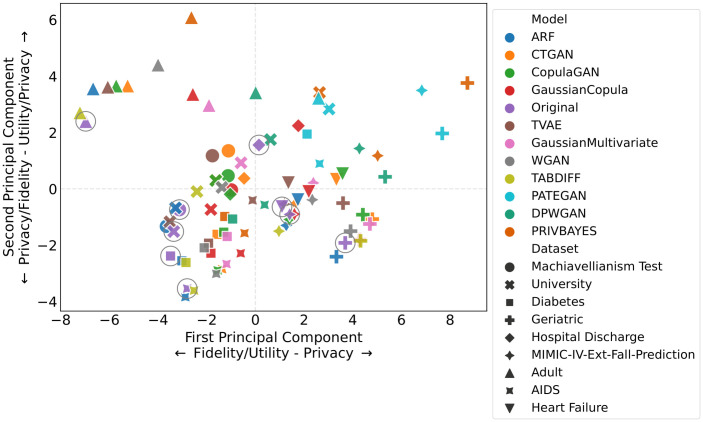
Trade-offs between privacy, utility, and fidelity as captured by the first (x-axis) and second (y-axis) principal component (PC) of variance of all metrics. On the x-axis (first PC), fidelity and utility metrics are negatively correlated with the component ← , while privacy metrics are positively correlated → , capturing the trade-off between (fidelity, utility) and privacy. On the y-axis (second PC), utility and the distance-related, inference and singling out privacy metrics correlate positively ↑ , whereas most fidelity and some privacy (against inference) metrics correlate negatively with the PC ↓ . The models form clusters associated with datasets in the principal component space, suggesting that variance related to datasets is larger than that related to model performance. The experiments ran on the Original data are circled for every dataset.

**Fig 5:**
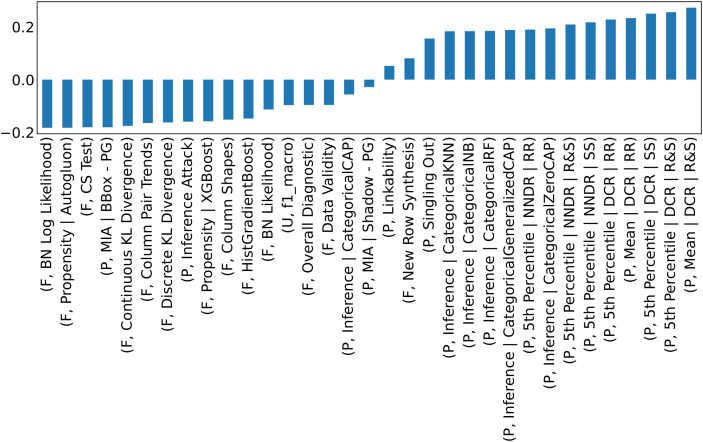
Trade-off between privacy and fidelity/utility as captured by the strongest variance direction - Principal Component (PC). The first PC is mainly positively correlated with privacy metrics, and mainly negatively correlated with fidelity and utility. For readability purposes, fidelity is shorten to F, utility is shorten to U and privacy is shorten to P.

**Fig 6:**
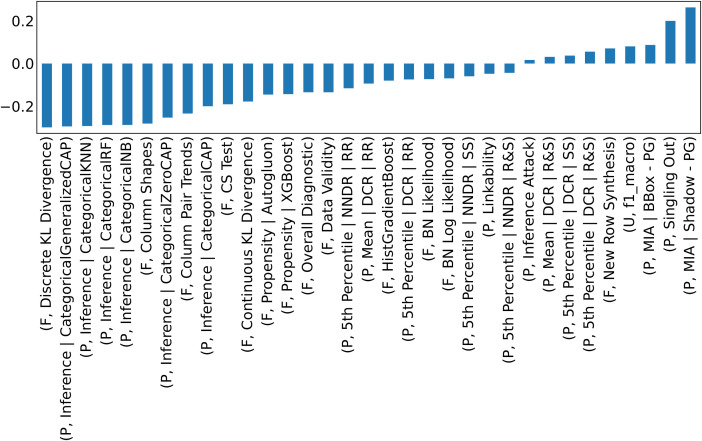
Trade-off between utility/fidelity and privacy. The second largest variance direction - PC, is mainly positively correlated with the utility metric, new row synthesis as fidelity metric, and privacy distanced-based metrics as well as singling out and inference attack, and mainly negatively correlated with the privacy PAI and distance-based metrics, and all other fidelity metrics like KL divergence and Propensity score. For readability purposes, fidelity is shorten to F, utility is shorten to U and privacy is shorten to P.

In real-world application scenarios for synthetic data, one of the most important questions is which generative model is optimal for a given use case. Our findings in Section 3.1 show that no single model excels in all metrics for any dataset. This implies that for every dataset a new model selection procedure needs to be performed. Finding the best model without actually evaluating all generative methods would be advantageous as it reduces the time to find the optimal generative model. Following this line of thought, we investigate how the variance of the metrics can be summarized by a linear low-dimensional manifold using Principal Component Analysis (PCA) on the 33 evaluation metrics scores to understand their importance on a 2-dimensional space (Fig 4). Our goal is to explore whether such analysis could help future generative model selection. We focus specifically on the first two Principal Components (PCs), which account for 45% of the total variance. To assess which aspects of individual metrics are captured by the main variance directions, we show the correlations between metrics and the first PC in Fig 5 (also x axis in Fig 4, and the second PC in Fig 6 (also y axis in Fig 4). This first PC already captures the main trade-off between privacy: most privacy metrics are positively correlated with the first PC, and most fidelity/utility are negatively correlated with the first PC. The second PC captures another aspect of the trade-off between utility and some privacy (inference attack, membership inference attack and singling out) metrics, which are positively correlated with the second PC, and all other privacy/fidelity metrics, which are negatively correlated with the second PC (Fig 6). Inspired by prior research [4,19], we investigate the trade-offs between fidelity/utility and privacy across different models and datasets. In Fig 4 the first and second PC are shown on the x and y axes respectively, illustrating how models and datasets behave along these dimensions. We notice that PATE-GAN, DP-WGAN and PrivBayes are mostly located on the positive direction of the first PC, ranked as the most private models. This is expected as PATE-GAN, DP-WGAN and PrivBayes are models that explicitly enforce differential privacy. Note that a similar DP mechanism could be added to all other models, too. What Fig 4 also shows is that in general, these models perform worse than all other models in terms of all non-privacy related metrics, hinting at the trade-off between utility and privacy.

Another aspect reflecting the results in Fig 4 is that within each dataset the models are clustered together. Some dataset clusters stand out, for example, Adult, for which models across the board often perform better in terms of fidelity and utility than in privacy (in the upper left quadrant); another example is the dataset from the Geriatric hospital in the lower right quadrant, with stronger privacy/fidelity metrics. Within every dataset ARF and TABDIFF are mostly on the left side, thus they rank better in terms of fidelity/utility. However, these observations require quantitative validation, which we provide in the following paragraph.

Can PCA be used to guide model selection? Evaluating all metrics on all models to find the optimal generative model might not always be feasible. The results in our PCA analysis suggest that maybe the much simpler trade-off between privacy and fidelity/utility as captured by the first PC allows to support model choices well enough. And computing the first PC from a subset of metrics to find the optimal generative model could increase computational efficiency.

To assess the potential of such a simplified trade-off, we consider two scenarios: a) a model selection where privacy is considered most important (e.g., cross-institutional or public compliant sharing of medical data) and b) a model selection procedure where utility/fidelity is prioritized (e.g., clinical model development within controlled environments). The first case a) could be one where patient data is shared outside a secure infrastructure. The second case b) could be one where the recipient of the synthetic data is a researcher who is tasked with developing the best ML model for a given task with the synthetic data. In case b) the data is not shared publicly and the individual researchers can be held accountable for their usage of the data.

To assess whether the first PC captures the trade-offs between all relevant metrics well enough to arrive at the same best performing model, we compare rankings derived from the first PC with those obtained from median metric scores (Table 4). For each dataset, models are ranked according to their first PC values (where higher scores indicate more private models) and by their median privacy and fidelity/utility scores, respectively. To quantify how similar the rankings obtained with the simpler first PC are to rankings obtained with the full suite of metrics, we computed Spearman’s rank correlation coefficient. We find high alignment between the simplified PCA based model selection compared to the model selection based on all metrics in 8 out of 9 (89%) of the datasets (Table 4) in terms of fidelity/utility and 5 out of 9 (55%) in terms of privacy. These results suggest that the simpler PCA can be a meaningful descriptive summary of the dominant trade-off between fidelity/utility and privacy scores for at least part of the datasets considered in our experiments, and may serve as a practical guideline for identifying models with favorable utility/fidelity characteristic. We further assume that evaluating a larger number of datasets could strengthen these observations.

**Table 4:** Spearman correlations between PC-based rankings and aggregated metric rankings. For every dataset we rank the models based on the 1st PC and a) on their median fidelity/utility metrics and b) median privacy metrics. We correlate these with the corresponding PC metrics and find that the correlations are high for the majority of the datasets, in particular 8 out of 9 data sets in the fidelity/utility use case; this indicates that the 1st PC alone yields model rankings similar to those computed on all metrics. Such correlations are less common for privacy.

Dataset	Rank Correlations with Priority on	
	(a) Fidelity/Utility	(b) Privacy
AIDS	0.95	0.77
Adult	0.94	0.90
Diabetes	0.94	0.27
Geriatric	0.87	0.59
Heart Failure	0.70	0.87
Hospital Discharge	1.00	-0.50
MIMIC-IV-Ext-Fall-Prediction	0.95	0.45
Machiavellianism Test	0.10	0.60
University	0.72	0.73

**Fig 7:**
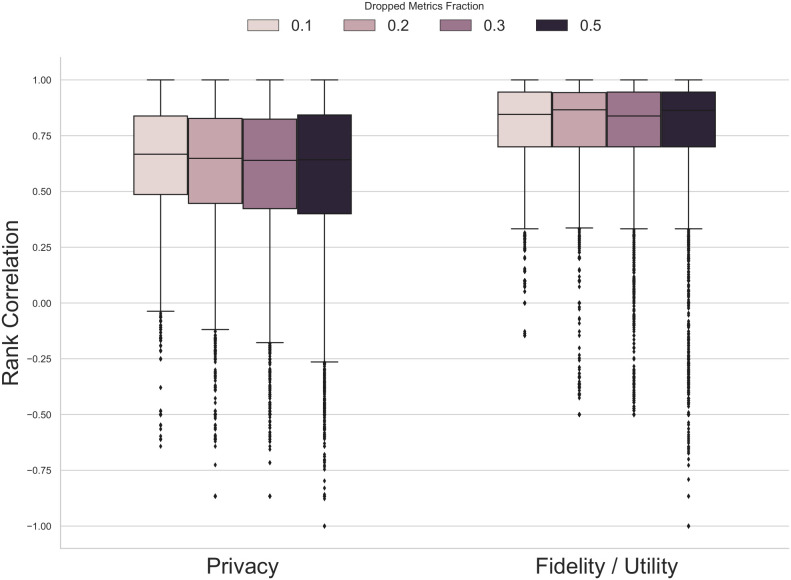
Spearman rank correlations between PC-based model rankings and rankings derived from aggregated median metric scores under metric subsampling. For each dataset, we perform 1,000 bootstrap runs removing 10%, 20%, 30%, and 50% of evaluation metrics, while always retaining the utility metric (F1-macro). Boxplots summarize the resulting correlation distributions.

To further assess the stability of PCA-based rankings for model selection, we conducted a bootstrap analysis with 1,000 resampling runs per dataset. We randomly removed 10%, 20%, 30%, and 50% of evaluation metrics while always retaining the utility score (represented by F1-macro), and again recomputed the PC-based and aggregated rankings (Fig 7). This assesses the PC-based rankings robustness with respect to different metrics sets.

Overall, PCA-based rankings exhibit strong stability under moderate metric subsampling, with most datasets maintaining high rank correlations even when up to 30% of metrics are removed. As the fraction of removed metrics increases, the variability of the rank correlations widens, reflected by a greater number of outliers and occasional negative correlations. We support these findings with the dataset-wise results in Fig B in S1 Appendix. We find that the stability of the correlations under different metric supsampling varies among datasets. Datasets such as AIDS, Adult, and Heart Failure remain highly robust even under 50% metric removal, whereas others, including Hospital Discharge, Machiavellianism Test, MIMIC-IV-Ext-Fall-Prediction and University, show increased sensitivity at higher metric removal rates.

An additional analysis includes regenerating and reevaluating synthetic data under multiple random seeds for two representative datasets - Adult and AIDS (Fig C and D in S1 Appendix). We perform the same PCA analysis on both datasets separately and observe that the first principal component again reflects the main trade-off between fidelity/utility in one direction and privacy in the opposite direction. Models such as DP-WGAN and PATEGAN are positioned more strongly along the privacy direction, whereas ARF and TABDIFF are more prominent on the fidelity/utility side, with the remaining models generally located in the middle. This observation in line with the main findings (Fig 4). Furthermore, we compute Spearman correlations between the first and second principal component loadings from the seed-based runs and the original loadings(Fig 5) and observe correlations of ρ≥0.65 for both datasets on the first principal loading, whereas the second loading shows lower correlation scores (Table R in S1 Appendix). The repeated experiments show model-wise stable behavior for most models. We observe some instabilities for DP-WGAN in both datasets, and some outliers for TVAE in AIDS.

Finally, we evaluate the sensitivity of the first PC rankings to different data normalizations. We recomputed the PCA using both Minmax and Z-score normalization and compared the resulting rankings to those obtained from the original PCA on the raw metric scores. Table 5 shows that the rankings remain highly correlated under different normalization strategies and that the PC-based rankings are not sensitive to metric scaling.

**Table 5:** Spearman correlations (median and 95% confidence interval) between PC-based model rankings computed from raw metric scores and those obtained after Min–Max and Z-score normalization. High correlations indicate that the rankings are largely insensitive to metric scaling.

Normalization method	rho (95% CI)
Minmax	0.94 (0.90, 1.00)
Z-score	0.94 (0.88, 1.00)

These findings suggest that the first PC can provide a meaningful descriptive summary of the main fidelity/utility and privacy trade-off for several of the datasets included in this study. Nevertheless, we also observe that in some dataset this rank alignment is weaker, indicating that depending on the application scenario, a more careful multi-metric evaluation may be necessary, especially in settings where privacy is of higher concern. Therefore, our PCA-based analysis provides a structured and transparent descriptive framework to summarize multi-metric trade-offs that could guide the initiation of model selection decisions.

### 3.3. Can dataset meta-features predict metrics?

Next we explore an alternative approach to guide model selection using dataset specific characteristics, or meta-features. This idea has also been investigated in the field of AutoML [60], where dataset meta-features have been used to explore the best ML models in predictive ML tasks. Here we follow a similar approach yet with simpler dataset meta-features. We investigate linear and non-linear dependencies between the dataset meta-features listed in Table 2 and all synthetic data metrics explored in our analyses. We perform the correlation analysis using the relative scores introduced in Section 2. This normalization removes dataset-specific effects, such as the difficulty of downstream task, measured with utility, to ensure that the correlations reflect the differences between the generative models rather than the datasets themselves.

For linear dependencies we computed correlation coefficients between the dataset meta-features and synthetic data metrics. We find no clear relationship between dataset features and generative model performance (Table 6).

**Table 6:** No dataset meta-feature is strongly correlated (ρ<0.5) with model performance across fidelity, utility, and privacy dimensions. Spearman correlations between dataset meta-features and the three evaluation metrics reveal only weak trends—fidelity shows mild positive associations with the number of features, percentage of boolean features, and class imbalance. Utility shows mild positive correlation with the number of samples, and the percentage of categorical features, and minor negative correlations with the number of features and the percentage of boolean features.

	Fidelity	Utility	Privacy
n_samples	0.04	0.36	-0.09
n_features	0.32	-0.24	-0.12
perc_num_features	-0.08	-0.10	-0.08
perc_bool_features	0.30	-0.13	0.10
perc_cat_features	-0.18	0.21	-0.14
class_imbalance	0.28	0.03	0.08

For non-linear dependencies, we used a decision tree model to evaluate how well synthetic dataset metrics can be predicted from non-linear combinations of dataset meta-features. We trained two models, one for predicting (a) best privacy generative model, and another for (b) best fidelity/utility generative model. The input features corresponded to the dataset meta-features, and the target labels for each dataset represented the best-performing generative model in terms of (a) privacy and (b) fidelity/utility. Labels were assigned by first aggregating (a) privacy metrics and then (b) fidelity/utility metrics within each dataset, ranking the models, and selecting the top-ranked one as the dataset label in both scenarios. Using these curated datasets, one with (a) privacy focused labels, and (b) fidelity/utility focused labels, we trained the decision trees with 5-fold cross-validation. In both cases, dataset meta-features were not predictive of synthetic dataset metrics, yielding median macro-F1 scores of 0.0 for the fidelity/utility-based model and 0.074 for the privacy-based model. These results suggest that in our experimental setting, dataset-level meta-features alone are not sufficient to reliably guide model selection. However, this analysis is statistically underpowered due to the limited number of datasets (*N* = 9) and the restricted set of simple meta-features considered. Therefore, the absence of predictive power in our experiments should not be interpreted as a general negative result on meta-learning for synthetic data model selection. More diverse meta-feature representations and a bigger dataset collection may provide an improved meta-feature-based selection guidance.

## 4. Discussion

Extending previous work [4,19], this study provides a comprehensive evaluation of synthetic tabular data methods including non-public, real-world patient datasets. In our analysis, we specifically explore trade-offs between fidelity, utility and privacy. Our empirical evaluation confirms that no single generative model outperforms all others across all three metrics. Instead, for each priority of metrics and each dataset, different models achieved the best synthetic data metrics. Our findings demonstrate that for every dataset and use case the optimal model needs to be found with a dedicated process, in close collaboration between all stakeholders, data engineers, ML engineers, data protection officers, and patient representatives.

We note that while it is tempting to ask for concrete thresholds of metrics that would allow for automated compliance tests of synthetic data, working on this study with data protection officers and lawyers we learned that it is not possible to provide more concrete technical guidance by means of thresholds of one or the other metric. We learned that it is the responsibility of researchers and engineers publishing data to continuously follow scientific trends and keep up with the latest state of the art in anonymization to make sure that synthetic data generation methods adhere to privacy regulations. Automated software components for synthetic data evaluation are key to these challenges. We hope that the process developed as part of this work and the software will help to guide the model optimization in this challenging setting. To support other researchers in this journey, we released our software package Synthius [36], also available on GitHub [61].

A key limitation of this study is the exclusive focus on single-table tabular data and classification tasks only. We do not consider other clinically relevant modalities such as medical imaging, clinical text, time-series data, or multi-modal settings. Extending the benchmark to these domains would require different model classes and evaluation criteria. To ensure comparability and reproducibility, we deliberately restricted the scope to tabular data and a unified evaluation framework. We therefore suggest that the identified model rankings and metric trade-offs should be interpreted as specific to the tabular setting.

Another limitation is the number of datasets (N = 9), which constrains the statistical power of our meta-feature analysis. We explored whether dataset characteristics such as number of samples, number of features, percentage of categorical or boolean features, and class imbalance can predict model performance. In line with the work of [62], we find that these meta-features alone do not reliably explain performance differences across generative models. Larger and more diverse dataset collections are needed to better characterize these relationships. Moreover, not all generative models could be successfully executed on every dataset due to computational constraints. For a small subset of datasets (Heart Failure, Machiavellianism Test, and Hospital Discharge), some models are therefore missing from the comparative evaluation. This reduces completeness for these datasets and may limit dataset-specific conclusions.

Given the diversity of generative model implementations and datasets evaluated in this study, a further limitation is that we do not perform a generative model-level MIA [19], which is computationally expensive for this given setting. Instead we use a black-box shadow model attack on downstream classifiers as a practical proxy for the privacy risk assessment, acknowledging that privacy risk estimates may differ from those obtained by directly attacking the generative model.

Our PCA analysis provides a quantifiable approach to navigate the trade-offs between the abundance of often conflicting metrics. Furthermore, sensitivity analyses under model and metric subsampling indicate that this structure is largely preserved under moderate perturbations, suggesting that the observed ranking patterns are not driven by a particular subset of models or evaluation metrics.

While our results can serve as an empirical basis for technical solutions to share synthetic healthcare data, further transdisciplinary work is needed to establish standardized evaluation frameworks to ensure that evaluation metrics are reflecting legal and ethical needs.

Overall, this work provides a structured and empirically driven framework for evaluating synthetic tabular data under competing metrics. Our results highlight that synthetic data evaluation is fundamentally a multi-objective optimization problem. Rather than searching for a universally superior model, systematic and robust evaluation frameworks are required to align generative models with specific use cases and stakeholder priorities. We consider this work as a step toward principled, automated, and transparent model selection strategies for synthetic data in healthcare. In this spirit, we hope that our results will also help navigate the trade-offs between privacy and fidelity/utility for researchers focusing generative AI in the future.

## Supporting information

S1 Appendix Supplementary tables and figures. (PDF)

S1 Data The publicly available datasets used in this study. (ZIP)
