# Supplementary material for: Evaluating the quality of tabular synthetic data in health care
Source: PLOS Digit Health. 2026 Jul 7;5(7):e0001522. doi: 10.1371/journal.pdig.0001522 (PMC13340858; doi:10.1371/journal.pdig.0001522)
Supplement: S1 Appendix — (PDF) [file pdig.0001522.s001.pdf]

S1 Appendix

Section A

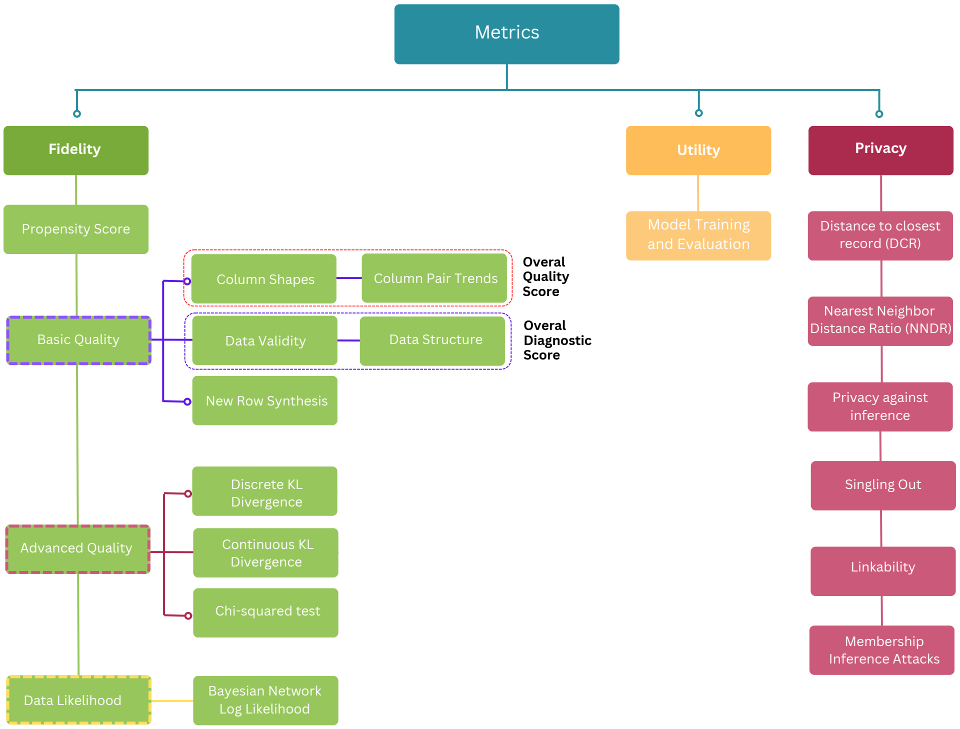

Fig A. The SDVMetrics hierarchy. We provide a visual representation of fidelity, utility and privacy and how they are structured within the SDVMetrics. Additionally, in the Privacy section we list the anonymeter metrics, Singling Out and Linkability.

Listing A. Filter criteria for fall patients applied on the MIMIC-IV-Ext-Fall-Prediction.

```
WHERE (
    long_title LIKE '%fall%'
) AND NOT (
    long_title LIKE '%fallopian%' OR
    long_title LIKE '%fallot%' OR
    long_title LIKE '%falling-object%' OR
    long_title LIKE '%watercraft%' OR
    long_title LIKE '%sport%' OR
    long_title LIKE '%ski%' OR
    long_title LIKE '%snowboard%' OR
    long_title LIKE '%mountain%' OR
    long_title LIKE '%cliff%' OR
    long_title LIKE '%aircraft%' OR
    long_title LIKE '%Animal%' OR
    long_title LIKE '%sailboat%' OR
    long_title LIKE '%swimming-pool%' OR
    long_title LIKE '%Striking%' OR
    long_title LIKE '%earth%' OR
```

```
long_title LIKE '%animal−rider%' OR
long_title LIKE '%Occupant%' OR
long_title LIKE '%fall-off%' OR
long_title LIKE '%train%' OR
long_title LIKE '%ice-and-snow%' OR
long_title LIKE '%without-falling%' OR
long_title LIKE '%Accidental%' OR
long_title LIKE '%ship%' OR
long_title LIKE '%boat%' OR
long_title LIKE '%kayak%' OR
long_title LIKE '%craft%'
);
```

Model	Dataset	Epsilon	Delta	Noise multiplier	Num Teachers	Teacher iters	Student iters	Epochs	lr (Generator)	lr (Discriminator)	Batch Size
PATEGAN	AIDS	1.30000	0.00001	0.001	N/1000	5	5	200	0.0001	0.0001	1024
PATEGAN	adult	1.50000	0.00001	0.001	N/1000	5	5	200	0.0001	0.0001	1024
PATEGAN	diabetes	1.50000	0.00001	0.001	N/1000	5	5	200	0.0001	0.0001	1024
PATEGAN	MIMIC	2.00000	0.00001	0.001	N/1000	5	5	200	0.0001	0.0001	1024
PATEGAN	University	1.30000	0.00001	0.001	N/1000	5	5	200	0.0001	0.0001	1024
PATEGAN	Geriatric	0.70000	0.00001	0.001	N/1000	5	5	200	0.0001	0.0001	1024
DPWGAN	AIDS	1.00000	0.00001	-	-	-	-	200	0.0002	0.0002	256
DPWGAN	adult	2.00000	0.00001	-	-	-	-	200	0.0002	0.0002	256
DPWGAN	diabetes	2.00000	0.00001	-	-	-	-	200	0.0002	0.0002	256
DPWGAN	MIMIC	0.70000	0.00001	-	-	-	-	200	0.0002	0.0002	256
DPWGAN	University	1.50000	0.00001	-	-	-	-	700	0.005	0.001	512
DPWGAN	Geriatric	1.30000	0.00001	-	-	-	-	700	0.005	0.001	512

Table A. Hyperparameters for PATEGAN and DPWGAN using the implementation provided by [1].

Category	Subcategory	Metric name (ours)	Metric name (others)	Used in
Basic Quality	Overall Quality	Column Shapes	Compare variable distributions	[2, 3]
Basic Quality	Overall Quality	Column Pair Trends	-	-
Basic Quality	Overall Diagnostic Score	Data validity	-	-
Basic Quality	Overall Diagnostic Score	Data structure	-	-
Basic Quality	-	New Row Synthesis	Novelty test	[4, 5]
Advanced Quality	-	Discrete KL Divergence	Pairwise Pearson correlation coefficient	[6, 7]
Advanced Quality	-	Continuous KL Divergence	-	-
Advanced Quality	-	Chi-squared test (CS Test)	Statistical tests	[3, 5]
Data Likelihood	-	Bayesian Network (BN) Likelihood	-	-
Data Likelihood	-	BN Log Likelihood	-	-
Propensity Score	-	Propensity Score	Discrimination ability, Domain classifier, Train an ML classifier to label data as real or synthetic	[8, 5, 7, 9]
-	-	-	Visualization techniques: principal component analysis (PCA), histograms and correlation matrices	[10, 3, 11, 9]

Table B. Comparison of the fidelity metrics with those used in other studies. Where "-" appears, no direct match exists between our defined metrics (ours) and those in prior research (others).

Metric name (ours)	Metric name (others)	Used in
Model Training and Evaluation	Model Compatibility, Machine Learning Efficacy, Application Fidelity	[12, 10, 13, 7, 9]

Table C. Comparison of the utility metrics with those used in other studies. Where "-" appears, no direct match exists between our defined metrics (ours) and those in prior research (others).

Metric name (ours)	Metric name (others)	Used in
Privacy Against Inference	Attribute disclosure	[14, 9]
DCR	DCR	[12, 11]
NNDR	NNDR	[11]
Singling out	Singling out	[9]
Linkability	Re-identification Attacks	[12]
-	Membership Inference attack	[12, 10, 9]
-	Identical Match Share (IMS)	[11]

Table D. Comparison of the privacy metrics with those used in other studies. Where "-" appears, no direct match exists between our defined metrics (ours) and those in prior research (others).

Section B

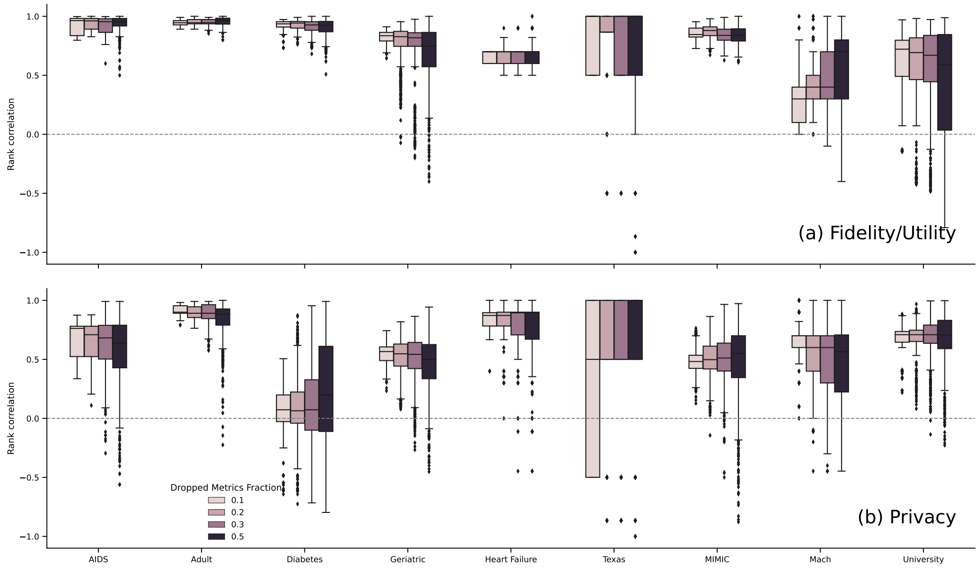

Fig B. Spearman rank correlations between PC-based model rankings and rankings derived from aggregated metric scores under metric subsampling. For each dataset, we perform 1,000 bootstrap runs removing 10%, 20%, 30%, and 50% of evaluation metrics, while always retaining the utility metric (f1_macro). Boxplots summarize the resulting correlation distributions. MIMIC-IV-Ext-Fall-Prediction, Machiavellianism Test and Hospital Discharge are abbreviated as MIMIC, Mach and Texas respectively. These results suggest that for most datasets, the PC-based rankings reflect a consistent evaluation pattern rather than being sensitive to the exact set of metrics used.

	Model	ARF	CTGAN	CopulaGAN	GaussianCopula	GaussianMultivariate	TVAE	WGAN	TABDIFF	PATEGAN	PRIVBAYES	DPWGAN
Dimension	Metric											
Utility	f1_macro	0.61	0.59	0.61	0.54	0.57	0.58	0.60	0.61	0.57	0.54	0.58
Fidelity	Column Pair Trends	0.96	0.86	0.87	0.88	0.88	0.77	0.90	0.74	0.53	0.69	0.72
	Column Shapes	0.99	0.93	0.92	0.94	0.94	0.82	0.94	0.99	0.64	0.94	0.80
	New Row Synthesis	1.00	1.00	1.00	1.00	1.00	1.00	1.00	1.00	1.00	1.00	1.00
	Overall Diagnostic	1.00	1.00	1.00	1.00	1.00	1.00	1.00	1.00	1.00	1.00	1.00
	CS Test	1.00	0.92	0.94	0.99	0.95	0.85	0.98	0.98	0.58	0.96	0.80
	Discrete KL Divergence	1.00	0.97	0.98	0.99	0.99	0.90	0.99	1.00	0.66	0.99	0.86
	Continuous KL Divergence	0.99	0.89	0.87	0.65	0.91	0.58	0.82	0.99	0.12	0.73	0.54
	BN Log Likelihood	-7.82	-8.29	-7.85	-8.36	-8.85	-5.89	-7.51	-7.54	-13.51	-8.54	-7.82
	Propensity — XGBoost	0.47	0.19	0.19	0.08	0.17	0.01	0.21	0.45	0.00	0.12	0.01
Privacy	5th Percentile — DCR — R&S	0.13	0.14	0.14	0.19	0.19	0.16	0.14	0.13	0.91	0.15	0.20
	5th Percentile — NNDR — R&S	0.57	0.57	0.58	0.60	0.58	0.85	0.60	0.54	0.79	0.58	0.70
	Inference — CategoricalCAP	1.00	1.00	0.95	1.00	1.00	0.96	1.00	1.00	1.00	0.00	1.00
	Singling Out	0.81	0.84	0.83	0.90	0.89	0.95	0.84	0.83	0.99	0.93	0.98
	Linkability	0.62	1.00	1.00	0.77	0.76	1.00	1.00	0.93	1.00	0.89	0.98
	Inference Attack	0.95	0.95	0.95	0.95	0.95	0.99	0.95	0.95	0.95	0.96	0.98
	MIA — Shadow - PG	0.31	0.31	0.31	0.32	0.31	0.95	0.31	0.31	0.35	0.32	0.48
	MIA — BBox - PG	0.34	0.21	0.34	0.08	0.20	0.60	0.36	0.36	0.17	0.22	0.40

Table E. AIDS dataset – metrics scores.

	Model	ARF	CTGAN	CopulaGAN	GaussianCopula	GaussianMultivariate	TVAE	WGAN	TABDIFF	PATEGAN	PRIVBAYES	DPWGAN
Dimension	Metric											
Utility	f1_macro	0.79	0.78	0.77	0.56	0.55	0.76	0.76	0.80	0.49	0.70	0.68
Fidelity	Column Pair Trends	0.98	0.85	0.88	0.81	0.78	0.87	0.80	0.98	0.34	0.69	0.44
	Column Shapes	1.00	0.90	0.93	0.88	0.87	0.91	0.90	0.99	0.47	0.71	0.66
	New Row Synthesis	0.98	1.00	1.00	1.00	1.00	1.00	1.00	1.00	1.00	1.00	1.00
	Overall Diagnostic	1.00	1.00	1.00	1.00	1.00	1.00	1.00	1.00	1.00	1.00	1.00
	CS Test	1.00	1.00	0.99	1.00	0.89	1.00	0.91	1.00	0.39	0.84	0.51
	Discrete KL Divergence	0.96	0.90	0.89	0.89	0.69	0.94	0.64	0.99	0.23	0.58	0.30
	Continuous KL Divergence	1.00	0.92	0.94	0.49	0.77	0.90	0.88	0.99	0.22	0.27	0.72
	BN Log Likelihood	-10.57	-10.86	-11.26	-14.97	-16.20	-8.50	-14.69	-9.05	-18.77	-14.46	-19.17
	Propensity — XGBoost	0.36	0.12	0.19	0.00	0.04	0.29	0.09	0.47	0.00	0.00	0.11
Privacy	5th Percentile — DCR — R&S	0.01	0.02	0.02	0.09	0.08	0.01	0.03	0.01	0.56	0.05	0.17
	5th Percentile — NNDR — R&S	0.26	0.40	0.38	0.63	0.57	0.41	0.46	0.30	0.73	0.53	0.69
	Inference — CategoricalCAP	0.33	0.40	0.36	0.53	0.59	0.33	0.33	0.33	1.00	0.33	0.81
	Singling Out	0.96	0.91	0.91	0.97	0.98	0.88	0.97	0.84	0.99	0.98	0.98
	Linkability	0.54	1.00	0.95	1.00	1.00	0.87	1.00	0.83	1.00	0.82	0.98
	Inference Attack	0.92	0.97	0.96	0.99	0.94	0.86	0.94	0.68	0.94	0.97	0.94
	MIA — Shadow - PG	0.77	0.77	0.77	0.78	0.78	0.80	0.78	0.77	0.78	0.77	0.78
	MIA — BBox - PG	0.83	1.17	1.14	0.82	0.20	1.10	0.82	0.87	0.20	0.59	0.59

Table F. Adult dataset – metrics scores.

	Model	ARF	CTGAN	CopulaGAN	GaussianCopula	GaussianMultivariate	TABDIFF	TVAE	WGAN	PATEGAN	PRIVBAYES	DPWGAN
Dimension	Metric											
Utility	f1_macro	0.67	0.63	0.57	0.58	0.58	0.68	0.63	0.62	0.50	0.58	0.60
Fidelity	Column Pair Trends	0.99	0.84	0.82	0.94	0.89	0.99	0.89	0.94	0.47	0.97	0.85
	Column Shapes	1.00	0.92	0.91	0.99	0.95	0.99	0.95	0.96	0.63	0.94	0.91
	New Row Synthesis	0.90	0.97	0.97	0.98	0.99	0.94	0.96	0.93	1.00	1.00	0.97
	Overall Diagnostic	1.00	1.00	1.00	1.00	1.00	1.00	1.00	1.00	1.00	1.00	1.00
	CS Test	1.00	0.91	0.89	0.99	0.94	0.99	0.96	0.94	0.44	0.99	0.92
	Discrete KL Divergence	1.00	0.94	0.92	0.99	0.97	1.00	0.96	0.94	0.52	1.00	0.88
	Continuous KL Divergence	1.00	0.89	0.88	0.92	0.77	0.99	0.94	0.96	0.39	0.90	0.84
	BN Log Likelihood	-13.39	-14.57	-14.65	-14.48	-15.72	-13.28	-13.06	-14.50	-27.59	-13.65	-16.51
	Propensity — XGBoost	0.48	0.18	0.15	0.21	0.15	0.45	0.22	0.26	0.00	0.00	0.08
Privacy	5th Percentile — DCR — R&S	0.00	0.01	0.02	0.02	0.04	0.00	0.01	0.01	0.46	0.03	0.02
	5th Percentile — NNDR — R&S	NaN	NaN	NaN	NaN	NaN	NaN	NaN	NaN	0.57	0.38	NaN
	Inference — CategoricalCAP	0.98	0.99	0.99	0.99	0.99	0.98	0.99	0.98	1.00	0.00	0.99
	Singling Out	0.96	0.89	0.89	0.88	0.90	0.94	0.90	0.90	0.94	0.93	0.93
	Linkability	0.84	1.00	1.00	0.99	0.99	1.00	0.99	1.00	1.00	1.00	1.00
	Inference Attack	0.96	0.96	0.96	0.96	0.96	0.96	0.96	0.96	0.97	0.96	0.95
	MIA — Shadow - PG	0.63	0.63	0.64	0.65	0.64	0.63	0.63	0.63	0.81	0.65	0.65
	MIA — BBox - PG	0.72	0.69	0.61	0.48	0.46	0.73	0.68	0.73	0.41	0.39	0.76

Table G. Diabetes dataset – metrics scores.

	Model	ARF	CTGAN	CopulaGAN	GaussianCopula	GaussianMultivariate	TVAE	WGAN	TABDIFF	PATEGAN	DPWGAN	PRIVBAYES
Dimension	Metric											
Utility	f1_macro	0.53	0.49	0.52	0.52	0.52	0.51	0.51	0.53	0.53	0.48	0.51
Fidelity	Column Pair Trends	0.97	0.83	0.84	0.93	0.88	0.83	0.85	0.88	0.49	0.71	0.53
	Column Shapes	0.99	0.91	0.91	0.97	0.93	0.89	0.92	0.97	0.65	0.82	0.76
	New Row Synthesis	1.00	1.00	1.00	1.00	1.00	1.00	1.00	1.00	1.00	1.00	1.00
	Overall Diagnostic	1.00	1.00	1.00	1.00	1.00	1.00	1.00	1.00	1.00	1.00	0.96
	CS Test	0.90	0.56	0.56	0.91	0.74	0.83	0.84	0.84	0.40	0.63	0.28
	Discrete KL Divergence	0.97	0.83	0.82	0.96	0.82	0.89	0.86	0.92	0.47	0.71	0.32
	Continuous KL Divergence	0.93	0.53	0.49	0.49	0.75	0.57	0.80	0.92	0.37	0.49	0.35
	BN Log Likelihood	-19.45	-18.63	-18.57	-19.11	-18.76	-26.23	-18.42	-23.86	-18.42	-18.42	NaN
	Propensity — XGBoost	0.28	0.01	0.01	0.01	0.02	0.14	0.05	0.01	0.00	0.00	0.00
Privacy	5th Percentile — DCR — R&S	1.47	1.58	1.44	1.58	1.74	1.24	1.58	2.01	3.37	2.00	2.66
	5th Percentile — NNDR — R&S	0.85	0.89	0.89	0.87	0.88	0.88	0.86	0.90	0.94	0.92	0.89
	Inference — CategoricalCAP	0.97	0.98	0.98	0.98	0.97	0.97	1.00	0.98	0.00	0.00	0.00
	Singling Out	0.95	0.97	0.97	0.96	0.95	0.98	0.94	0.95	0.99	0.98	0.97
	Linkability	1.00	1.00	1.00	1.00	1.00	1.00	1.00	1.00	1.00	1.00	1.00
	Inference Attack	0.25	0.25	0.25	0.25	0.25	0.25	0.25	0.25	0.25	0.25	0.25
	MIA — Shadow - PG	0.46	0.45	0.48	0.46	0.49	0.94	0.48	0.46	0.98	0.65	0.90
	MIA — BBox - PG	0.46	0.31	0.57	0.28	0.43	0.74	0.60	0.30	0.02	0.58	0.00

Table H. Geriatric Fall Prediction dataset – metrics scores.

Dimension	Model<br>Metric	ARF	CTGAN	CopulaGAN	GaussianCopula	TVAE
Utility	f1_macro	0.88	0.45	0.37	**0.96**	0.93
Fidelity	Column Pair Trends	0.84	0.78	0.76	**0.88**	0.65
	Column Shapes	**0.97**	0.84	0.83	0.94	0.85
	New Row Synthesis	**1.00**	**1.00**	**1.00**	**1.00**	**1.00**
	Overall Diagnostic	1.00	**1.00**	**1.00**	**1.00**	**1.00**
	CS Test	0.72	0.64	0.66	0.73	**0.76**
	Discrete KL Divergence	**0.72**	0.49	0.36	0.69	0.71
	Continuous KL Divergence	nan	nan	nan	nan	nan
	BN Log Likelihood	0.00	0.00	0.00	0.00	**0.00**
	Propensity — XGBoost	**0.06**	0.02	0.00	0.00	0.03
Privacy	5th Percentile — DCR — R&S	0.74	1.19	**1.22**	1.07	0.62
	5th Percentile — NNDR — R&S	0.72	0.81	**0.83**	0.81	0.78
	Inference — CategoricalCAP	-35.17	-38.50	-51.74	-39.83	**-28.48**
	Inference — CategoricalEnsemble	**0.00**	**0.00**	**0.00**	**0.00**	**0.00**
	Singling Out	0.91	0.96	**1.00**	0.97	0.99
	Linkability	0.99	**1.00**	1.00	**1.00**	**1.00**

Table I. Heart Failure dataset – metrics scores.

Dimension	Model<br>Metric	CTGAN	CopulaGAN	GaussianCopula
Utility	f1_macro	**1.00**	**1.00**	**1.00**
Fidelity	Column Pair Trends	0.86	0.87	**0.88**
	Column Shapes	0.84	**0.86**	0.76
	New Row Synthesis	**1.00**	**1.00**	**1.00**
	Overall Diagnostic	**1.00**	**1.00**	0.97
	CS Test	0.95	**0.96**	0.90
	Discrete KL Divergence	0.96	**0.98**	0.70
	Continuous KL Divergence	nan	nan	nan
	BN Log Likelihood	**0.00**	0.00	0.00
	Propensity — XGBoost	0.00	**0.54**	0.00
Privacy	5th Percentile — DCR — R&S	1.25	1.25	**1.52**
	5th Percentile — NNDR — R&S	**0.96**	0.96	0.95
	Inference — CategoricalCAP	**-57.34**	-59.75	-80.17
	Inference — CategoricalEnsemble	**0.00**	**0.00**	**0.00**
	Singling Out	0.98	0.98	**1.00**

Table J. Hospital Discharge dataset – metrics scores.

Dimension	Model<br>Metric	ARF	CTGAN	CopulaGAN	GaussianCopula	GaussianMultivariate	TVAE	WGAN	TABDIFF	PATEGAN	DPWGAN	PRIVBAYES
Utility	f1_macro	0.57	0.55	0.56	0.52	0.53	0.57	0.63	0.64	0.50	0.55	0.47
Fidelity	Column Pair Trends	0.93	0.71	0.74	0.79	0.65	0.65	0.65	0.92	0.42	0.60	0.26
	Column Shapes	0.98	0.89	0.90	0.92	0.86	0.87	0.82	0.97	0.52	0.71	0.77
	New Row Synthesis	1.00	1.00	1.00	1.00	1.00	1.00	1.00	1.00	1.00	1.00	1.00
	Overall Diagnostic	1.00	1.00	1.00	1.00	0.99	1.00	1.00	1.00	0.99	0.99	1.00
	CS Test	1.00	0.94	0.97	1.00	0.93	0.95	0.99	0.98	0.30	0.70	0.91
	Discrete KL Divergence	0.89	0.78	0.79	0.87	0.69	0.77	0.61	0.89	0.24	0.48	0.86
	Continuous KL Divergence	0.99	0.98	0.96	0.96	0.91	0.97	0.96	0.99	0.28	0.61	0.23
	BN Log Likelihood	-18.72	-19.26	-19.74	-18.49	-18.42	-19.84	-18.42	-19.08	-18.42	-18.42	-36.05
	Propensity — XGBoost	0.00	0.00	0.00	0.00	0.00	0.00	0.00	0.00	0.00	0.00	0.00
Privacy	5th Percentile — DCR — R&S	0.48	0.49	0.45	0.52	0.53	0.39	1.09	0.42	2.53	1.31	0.97
	5th Percentile — NNDR — R&S	0.71	0.73	0.73	0.72	0.73	0.78	0.83	0.71	0.89	0.87	0.78
	Inference — CategoricalCAP	0.00	0.00	1.00	0.00	0.00	1.00	1.00	0.97	0.00	0.00	0.00
	Singling Out	0.97	0.98	0.98	0.98	0.99	0.95	0.99	1.00	1.00	0.99	0.97
	Linkability	1.00	0.96	0.80	0.80	0.95	1.00	0.78	0.87	0.46	1.00	NaN
	Inference Attack	0.54	0.59	0.54	0.54	0.54	0.65	0.92	0.54	0.25	0.25	0.31
	MIA — Shadow - PG	0.50	0.49	0.49	0.50	0.58	0.74	0.49	0.49	0.53	0.70	0.78
	MIA — BBox - PG	0.49	0.62	0.51	0.39	0.34	0.72	0.44	0.59	0.22	0.29	0.05

Table K. MIMIC-IV-Ext-Fall-Prediction dataset – metrics scores.

Dimension	Model<br>Metric	ARF	CTGAN	CopulaGAN	GaussianCopula	TVAE
Utility	f1_macro	0.50	0.54	**0.56**	0.50	0.50
Fidelity	Column Pair Trends	**0.99**	0.80	0.80	0.88	0.72
	Column Shapes	**1.00**	0.89	0.88	0.96	0.88
	New Row Synthesis	0.99	0.99	1.00	**1.00**	0.99
	Overall Diagnostic	**1.00**	**1.00**	**1.00**	**1.00**	**1.00**
	CS Test	**1.00**	0.30	0.31	1.00	0.99
	Discrete KL Divergence	**1.00**	0.87	0.91	0.83	0.77
	Continuous KL Divergence	**1.00**	0.83	0.83	0.95	0.86
	BN Log Likelihood	-15.84	-15.97	-16.24	-18.13	**-14.29**
	Propensity — XGBoost	**0.45**	0.18	0.16	0.12	0.05
Privacy	5th Percentile — DCR — R&S	0.09	0.12	0.11	**0.28**	0.10
	5th Percentile — NNDR — R&S	0.57	0.67	0.66	**0.73**	0.73
	Inference — CategoricalCAP	0.78	0.78	**0.79**	0.79	0.75
	Inference — CategoricalEnsemble	0.71	0.75	**0.76**	0.75	0.73
	Singling Out	0.82	**1.00**	0.87	0.98	0.95
	Linkability	1.00	**1.00**	**1.00**	**1.00**	1.00

Table L. Machiavellianism Test dataset – metrics scores.

Dimension	Model<br>Metric	ARF	CTGAN	CopulaGAN	GaussianCopula	GaussianMultivariate	TVAE	WGAN	TABDIFF	PATEGAN	DPWGAN	PRIVBAYES
Utility	f1_macro	0.53	0.51	0.50	0.50	0.51	0.50	0.55	0.54	0.54	0.54	0.53
Fidelity	Column Pair Trends	0.99	0.75	0.75	0.93	0.84	0.95	0.88	0.92	0.40	0.62	0.28
	Column Shapes	0.99	0.90	0.89	0.99	0.90	0.97	0.94	0.97	0.56	0.73	0.58
	New Row Synthesis	0.61	0.93	0.94	1.00	1.00	0.66	1.00	0.99	1.00	1.00	1.00
	Overall Diagnostic	1.00	1.00	1.00	1.00	0.99	1.00	1.00	1.00	1.00	1.00	0.99
	CS Test	0.98	0.73	0.74	0.97	0.84	0.96	0.97	0.97	0.19	0.44	0.11
	Discrete KL Divergence	0.94	0.76	0.75	0.82	0.69	0.92	0.78	0.81	0.26	0.45	0.23
	BN Log Likelihood	-11.96	-15.43	-15.98	-15.62	-18.12	-10.98	-16.60	-12.51	-18.42	-18.32	-18.41
	Propensity — XGBoost	0.00	0.00	0.00	0.00	0.00	0.00	0.00	0.00	0.00	0.00	0.00
Privacy	5th Percentile — DCR — R&S	0.00	0.00	0.00	0.00	0.00	0.00	0.01	0.00	1.58	0.08	0.02
	5th Percentile — NNDR — R&S	0.17	0.20	0.22	0.24	0.24	0.23	0.15	0.16	0.93	0.37	0.28
	Inference — CategoricalCAP	0.91	0.98	0.98	0.99	0.97	0.92	0.96	0.95	0.98	0.97	0.99
	Singling Out	0.98	0.95	0.96	0.90	0.97	0.83	0.94	1.00	1.00	1.00	0.99
	Linkability	0.94	0.95	1.00	1.00	0.90	0.94	0.98	0.96	1.00	1.00	1.00
	Inference Attack	0.22	0.68	0.80	0.38	0.38	0.25	0.25	0.68	0.91	0.77	0.80
	MIA — Shadow - PG	0.94	0.94	0.94	0.95	0.94	0.95	0.95	0.95	0.98	0.96	0.98
	MIA — BBox - PG	0.97	0.99	0.99	0.96	0.86	0.99	0.92	0.95	0.86	0.99	0.18

Table M. University Fall Prediction dataset – metrics scores.

Metric	Model A	Model B	p-value
Fidelity	TABDIFF	PATEGAN	0.000
Fidelity	ARF	PATEGAN	0.000
Fidelity	TABDIFF	DPWGAN	0.020
Fidelity	GaussianCopula	PATEGAN	0.022
Fidelity	TABDIFF	PRIVBAYES	0.024
Fidelity	ARF	DPWGAN	0.025
Fidelity	ARF	PRIVBAYES	0.029
Fidelity	WGAN	PATEGAN	0.134
Fidelity	TVAE	PATEGAN	0.308
Fidelity	CTGAN	PATEGAN	0.370
Fidelity	CopulaGAN	PATEGAN	0.436
Fidelity	GaussianCopula	DPWGAN	0.659
Fidelity	GaussianCopula	PRIVBAYES	0.741
Fidelity	GaussianMultivariate	PATEGAN	0.959
Fidelity	TVAE	WGAN	1.000
Fidelity	TVAE	GaussianMultivariate	1.000
Fidelity	TVAE	TABDIFF	1.000
Fidelity	TVAE	DPWGAN	1.000
Fidelity	ARF	CTGAN	1.000

Table N. Model-level pairwise Dunn test with Bonferroni corrections ($\alpha < 0.05$) applied to the synthetic absolute scores for each metric (fidelity, utility, and privacy). The test is conducted across seven models, with each model represented by a vector of the metric scores from all nine datasets. For better clarity, only a small selection of all pairs (most of which have the same p-value of 1.0) are displayed.

Metric	Model A	Model B	p-value
Fidelity	ARF	PATEGAN	0.006
Fidelity	TABDIFF	PATEGAN	0.012
Privacy	ARF	GaussianCopula	0.013
Privacy	ARF	PATEGAN	0.026
Fidelity	ARF	DPWGAN	0.047
Privacy	ARF	PRIVBAYES	0.076
Fidelity	TABDIFF	DPWGAN	0.079
Privacy	ARF	CopulaGAN	0.083
Privacy	ARF	DPWGAN	0.088
Fidelity	TVAE	PATEGAN	0.137
Utility	GaussianMultivariate	TABDIFF	0.186
Utility	TABDIFF	PRIVBAYES	0.193
Fidelity	GaussianCopula	PATEGAN	0.212
Privacy	ARF	CTGAN	0.238
Utility	TABDIFF	PATEGAN	0.323
Fidelity	ARF	PRIVBAYES	0.352
Fidelity	TABDIFF	PRIVBAYES	0.488
Utility	GaussianCopula	TABDIFF	0.520
Privacy	ARF	GaussianMultivariate	0.526
Utility	TABDIFF	DPWGAN	0.608
Fidelity	TVAE	DPWGAN	0.716
Privacy	ARF	WGAN	0.742
Privacy	GaussianCopula	TABDIFF	0.782

Privacy	TABDIFF	PATEGAN	0.848
Fidelity	WGAN	PATEGAN	0.994
Utility	ARF	PRIVBAYES	1.000
Utility	CTGAN	CopulaGAN	1.000

Table O. Model-level pairwise Dunn test with Bonferroni corrections ($\alpha < 0.05$) applied to the synthetic relative scores for each metric (fidelity, utility, and privacy). The test is conducted across seven models, with each model represented by a vector of the metric scores from all nine datasets. For better clarity, only statistically significant different pairs (p-value < 0.05) and a small selection of the other pairs (all of which have the same p-value of 1.0) are displayed.

Metric	Dataset A	Dataset B	p-value
Privacy	Geriatric	AIDS	0.000
Privacy	Geriatric	Adult	0.000
Privacy	University	Adult	0.000
Privacy	Adult	Heart Failure	0.000
Utility	Geriatric	Adult	0.002
Utility	Geriatric	Hospital Discharge	0.003
Privacy	AIDS	Heart Failure	0.005
Privacy	Machiavellianism Test	Geriatric	0.008
Utility	University	Hospital Discharge	0.009
Utility	University	Adult	0.013
Utility	Machiavellianism Test	Hospital Discharge	0.017
Privacy	Geriatric	MIMIC-IV-Ext-Fall-Prediction	0.017
Utility	Diabetes	Geriatric	0.026
Privacy	Hospital Discharge	Adult	0.032
Privacy	Diabetes	Adult	0.039
Fidelity	University	Diabetes	0.046
Privacy	Machiavellianism Test	Heart Failure	0.055
Fidelity	Diabetes	Heart Failure	0.057
Utility	Machiavellianism Test	Adult	0.064
Utility	Geriatric	AIDS	0.088
Privacy	University	AIDS	0.100
Utility	University	Diabetes	0.113
Privacy	Diabetes	Geriatric	0.131
Fidelity	Diabetes	MIMIC-IV-Ext-Fall-Prediction	0.139
Utility	Hospital Discharge	MIMIC-IV-Ext-Fall-Prediction	0.159
Privacy	MIMIC-IV-Ext-Fall-Prediction	Heart Failure	0.200
Fidelity	Diabetes	Geriatric	0.240
Privacy	MIMIC-IV-Ext-Fall-Prediction	Adult	0.261
Utility	Machiavellianism Test	Diabetes	0.297
Utility	University	AIDS	0.334
Privacy	Hospital Discharge	AIDS	0.555
Utility	Machiavellianism Test	AIDS	0.654
Utility	MIMIC-IV-Ext-Fall-Prediction	Adult	0.745
Privacy	Diabetes	Heart Failure	0.751
Privacy	Machiavellianism Test	University	0.830
Fidelity	AIDS	Heart Failure	0.989
Privacy	MIMIC-IV-Ext-Fall-Prediction	AIDS	1.000
Utility	Hospital Discharge	Heart Failure	1.000
Utility	Diabetes	Hospital Discharge	1.000

Utility	MIMIC-IV-Ext-Fall-Prediction	AIDS	1.000

Table P. Data-level pairwise Dunn test with Bonferroni corrections ($\alpha < 0.05$) applied to the synthetic absolute scores for each metric (fidelity, utility, and privacy). The test is conducted across nine datasets, with each dataset represented by a vector of metric scores from all seven models. The results are sorted by the p-value in ascending manner. For better clarity, only statistically significant different pairs (p-value < 0.05) and a small selection of the other pairs (all of which have the same p-value of 1.0) are displayed. We refer to MIMIC-IV-Ext-Fall-Prediction as MIMIC for better readability.

Metric	Dataset A	Dataset B	p-value
Privacy	Adult	AIDS	0.001
Fidelity	MIMIC-IV-Ext-Fall-Prediction	Adult	0.001
Fidelity	Hospital Discharge	Adult	0.012
Utility	Hospital Discharge	MIMIC-IV-Ext-Fall-Prediction	0.014
Privacy	University	AIDS	0.044
Utility	University	MIMIC-IV-Ext-Fall-Prediction	0.044
Fidelity	MIMIC-IV-Ext-Fall-Prediction	AIDS	0.046
Utility	Geriatric	Hospital Discharge	0.078
Fidelity	Diabetes	MIMIC-IV-Ext-Fall-Prediction	0.084
Fidelity	Machiavellianism Test	MIMIC-IV-Ext-Fall-Prediction	0.119
Fidelity	Hospital Discharge	AIDS	0.133
Privacy	MIMIC-IV-Ext-Fall-Prediction	Adult	0.143
Fidelity	Machiavellianism Test	Hospital Discharge	0.144
Privacy	Diabetes	Adult	0.151
Privacy	Hospital Discharge	AIDS	0.169
Fidelity	Diabetes	Hospital Discharge	0.191
Utility	MIMIC-IV-Ext-Fall-Prediction	AIDS	0.215
Utility	Hospital Discharge	Heart Failure	0.226
Utility	Hospital Discharge	Adult	0.268
Utility	University	Geriatric	0.437
Utility	Machiavellianism Test	MIMIC-IV-Ext-Fall-Prediction	0.451
Fidelity	University	Adult	0.456
Fidelity	Geriatric	MIMIC-IV-Ext-Fall-Prediction	0.591
Fidelity	Geriatric	Hospital Discharge	0.648
Utility	Diabetes	Hospital Discharge	0.750
Privacy	Geriatric	Adult	0.861
Utility	Machiavellianism Test	University	1.000
Privacy	Adult	Heart Failure	1.000
Utility	Machiavellianism Test	Adult	1.000

Table Q. Data-level pairwise Dunn test with Bonferroni corrections ($\alpha < 0.05$) applied to the synthetic relative scores for each metric (fidelity, utility, and privacy). The test is conducted across nine datasets, with each dataset represented by a vector of scores from all seven models. The results are sorted by the p-value in ascending manner. For better clarity, only statistically significant different pairs (p-value < 0.05) and a small selection of the other pairs (all of which have the same p-value of 1.0) are displayed. We refer to MIMIC-IV-Ext-Fall-Prediction as MIMIC for better readability.

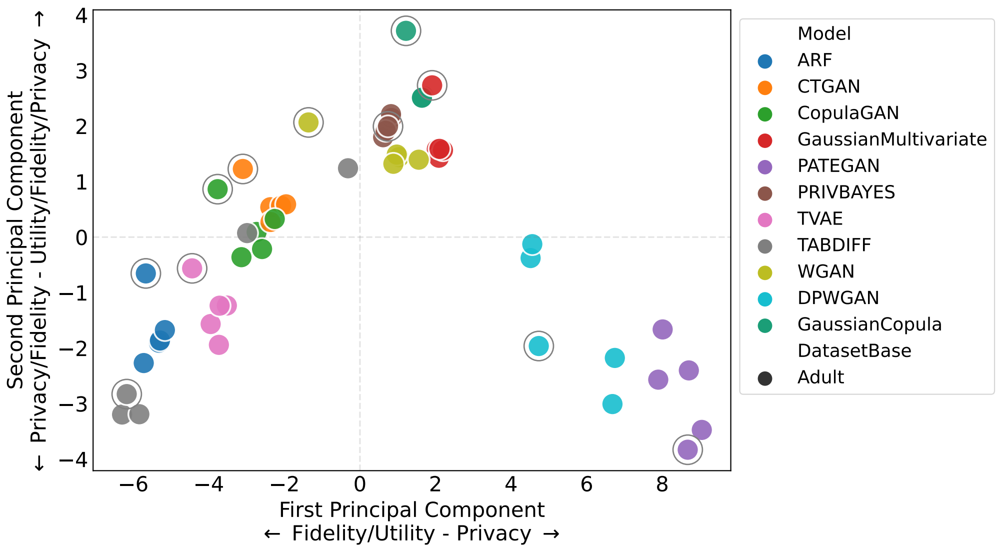

Fig C. PCA on different runs (synthesized data) under different random seeds for the Adult dataset. The first PC is a trade-off between fidelity and utility in the negative direction and privacy in the positive direction. The circled data points are the original synthetic data used in the main analysis of the study.

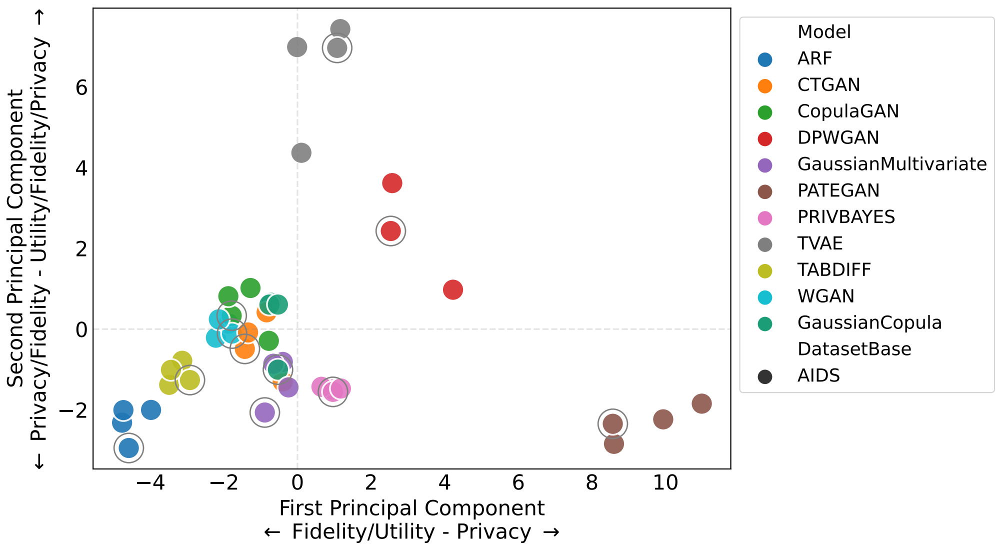

Fig D. PCA on different runs (synthesized data) under different random seeds for the AIDS dataset. The first PC is a trade-off between fidelity and utility in the negative direction and privacy in the positive direction. The circled data points are the original synthetic data used in the main analysis of the study.

Loading	Comparison	Spearman (ρ)	p-value
L1	Original - Adult	**0.735**	0.000001
L1	Original - AIDS	**0.668**	0.000022
L1	Adult - AIDS	**0.822**	4.500000e-09
L2	Original - Adult	0.242	0.174524
L2	Original - AIDS	0.593	0.00028
L2	Adult - AIDS	0.272	0.126301

Table R. Correlations between the first (L1) and second (L2) PC loadings between the different runs for Adult (Fig C and AIDS (Fig D). Both datasets show strong correlations ($\rho_s \geq 0.65$) with the original first Principal Component (L1: Original - Adult and Original - AIDS).

References

1. Truda G. Generating tabular datasets under differential privacy. arXiv preprint arXiv:230814784. 2023.

2. Che Z, Cheng Y, Zhai S, Sun Z, Liu Y. Boosting Deep Learning Risk Prediction with Generative Adversarial Networks for Electronic Health Records. In: 2017 IEEE International Conference on Data Mining (ICDM). IEEE; 2017. p. 787–792. Available from: `http://dx.doi.org/10.1109/ICDM.2017.93`. doi:10.1109/icdm.2017.93.

3. Bourou S, El Saer A, Velivassaki TH, Voulkidis A, Zahariadis T. A Review of Tabular Data Synthesis Using GANs on an IDS Dataset. Information. 2021 Sep;12(9):375. Available from: `http://dx.doi.org/10.3390/info12090375`. doi:10.3390/info12090375.

4. Herurkar D, Sattarov T, Hees J, Palacio S, Raue F, Dengel A. Cross-Domain Transformation for Outlier Detection on Tabular Datasets. In: 2023 International Joint Conference on Neural Networks (IJCNN). IEEE; 2023. p. 1–8. Available from: `http://dx.doi.org/10.1109/IJCNN54540.2023.10191326`. doi:10.1109/ijcnn54540.2023.10191326.

5. Livieris IE, Alimpertis N, Domalis G, Tsakalidis D. An evaluation framework for synthetic data generation models. arXiv; 2024. Available from: `https://arxiv.org/abs/2404.08866`. doi:10.48550/ARXIV.2404.08866.

6. Wang L, Zhang W, He X. In: Continuous Patient-Centric Sequence Generation via Sequentially Coupled Adversarial Learning. Springer International Publishing; 2019. p. 36–52. Available from: `$http://dx.doi.org/10.1007/978-3-030-18579-4_3$`. doi:10.1007/$978 - 3 - 030 - 18579 - 4_3$.

7. Dankar FK, Ibrahim MK, Ismail L. A Multi-Dimensional Evaluation of Synthetic Data Generators. IEEE Access. 2022;10:11147–11158. Available from: `http://dx.doi.org/10.1109/ACCESS.2022.3144765`. doi:10.1109/access.2022.3144765.

8. Spadotto T, Toldo M, Michieli U, Zanuttigh P. Unsupervised Domain Adaptation with Multiple Domain Discriminators and Adaptive Self-Training. In: 2020 25th International Conference on Pattern Recognition (ICPR). IEEE; 2021. p. 2845–2852. Available from:

http://dx.doi.org/10.1109/ICPR48806.2021.9412894. doi:10.1109/icpr48806.2021.9412894.

9. Adams T, Birkenbihl C, Otte K, Ng HG, Rieling JA, Näher AF, et al. On the Trade-Off between Fidelity, Utility and Privacy of Synthetic Patient Data. 2024 Dec. Available from: http://dx.doi.org/10.1101/2024.12.06.24317239. doi:10.1101/2024.12.06.24317239.

10. Yale A, Dash S, Dutta R, Guyon I, Pavao A, Bennett KP. Generation and evaluation of privacy preserving synthetic health data. Neurocomputing. 2020 Nov;416:244–255. Available from: http://dx.doi.org/10.1016/j.neucom.2019.12.136. doi:10.1016/j.neucom.2019.12.136.

11. D'Amico S, Dall'Olio D, Sala C, Dall'Olio L, Sauta E, Zampini M, et al. Synthetic Data Generation by Artificial Intelligence to Accelerate Research and Precision Medicine in Hematology. JCO Clinical Cancer Informatics. 2023 Jun;(7). Available from: http://dx.doi.org/10.1200/CCI.23.00021. doi:10.1200/cci.23.00021.

12. Park N, Mohammadi M, Gorde K, Jajodia S, Park H, Kim Y. Data synthesis based on generative adversarial networks. Proceedings of the VLDB Endowment. 2018 Jun;11(10):1071–1083. Available from: http://dx.doi.org/10.14778/3231751.3231757. doi:10.14778/3231751.3231757.

13. El Emam K, Mosquera L, Fang X, El-Hussuna A. Utility Metrics for Evaluating Synthetic Health Data Generation Methods: Validation Study. JMIR Medical Informatics. 2022 Apr;10(4):e35734. Available from: http://dx.doi.org/10.2196/35734. doi:10.2196/35734.

14. Choi E, Biswal S, Malin B, Duke J, Stewart WF, Sun J. Generating Multi-label Discrete Patient Records using Generative Adversarial Networks. In: Doshi-Velez F, Fackler J, Kale D, Ranganath R, Wallace B, Wiens J, editors. Proceedings of the 2nd Machine Learning for Healthcare Conference. vol. 68 of Proceedings of Machine Learning Research. PMLR; 2017. p. 286-305. Available from: https://proceedings.mlr.press/v68/choi17a.html.
